# AcMYB1 Interacts With AcbHLH1 to Regulate Anthocyanin Biosynthesis in *Aglaonema commutatum*

**DOI:** 10.3389/fpls.2022.886313

**Published:** 2022-07-19

**Authors:** Ji Li, Kunlin Wu, Lin Li, Guohua Ma, Lin Fang, Songjun Zeng

**Affiliations:** ^1^Key Laboratory of South China Agricultural Plant Molecular Analysis and Gene Improvement, South China Botanical Garden, Chinese Academy of Sciences, Guangzhou, China; ^2^University of Chinese Academy of Sciences, Beijing, China; ^3^Guangdong Provincial Key Laboratory of Applied Botany, South China Botanical Garden, Chinese Academy of Sciences, Guangzhou, China; ^4^Center of Economic Botany, Core Botanical Gardens, Chinese Academy of Sciences, Guangzhou, China

**Keywords:** *Aglaonema commutatum* “Red Valentine”, anthocyanin pathway, R2R3-MYB, transcriptional regulation, transgenic tobacco

## Abstract

*Aglaonema commutatum* is one of the most popular foliage plants with abundant leaf phenotypes; therefore, anthocyanin coloration is a vital economic trait in *A. commutatum*. However, the molecular mechanisms underlying anthocyanin biosynthesis and its regulation remain unclear. In this study, *AcMYB1* and *AcbHLH1*, transcription factor genes related to an R2R3-myeloblast (MYB) and a basic helix–loop–helix (bHLH), respectively, were isolated from *A. commutatum* “Red Valentine” and functionally characterized. AcMYB1 and AcbHLH1 were found to interact by Y2H and BiFC assay. *AcMYB1* was grouped into the AN2 subgroup and shared high homology with the known regulators of anthocyanin biosynthesis. Gene expression analysis showed that both *AcMYB1* and *AcbHLH1* have similar expression patterns to anthocyanin structural genes and correlate with anthocyanin distribution in different tissues of *A. commutatum*. Light strongly promoted anthocyanin accumulation by upregulating the expression of anthocyanin-related genes in *A. commutatum* leaves. Ectopic expression of *AcMYB1* in tobacco remarkably increased anthocyanin accumulation in both vegetative and reproductive tissues at various developmental stages. These results provide insights into the regulation of anthocyanin biosynthesis in *A. commutatum* and are useful for breeding new *A. commutatum* cultivars with enhanced ornamental value.

## Introduction

Anthocyanins are broadly located in plant species and are responsible for a wide range of coloration, such as purple, blue, and pink, in plant flowers, fruits, and leaves (Winkel-Shirley, 2001). Anthocyanins are vital secondary metabolites that attract insect pollinators and help defend plants against biotic and abiotic stresses (Schaefer et al., 2004). In vegetative organs, anthocyanins act as a barrier to protect photosynthetic cells from intense light (Hughes et al., 2005). More importantly, there is growing evidence that anthocyanins are beneficial to human health by reducing the rates of cardiovascular disease, obesity, diabetes, lung disease, and cancer (Hou, 2003; Martin et al., 2011; Ha et al., 2015).

The biochemistry and enzymology of the anthocyanin pathway are among the most widely studied pathways in plant secondary metabolites, and almost all encoding enzymes have been isolated and characterized (Mol et al., 1998; Koes et al., 2005). Chalcone synthase (*CHS*), chalcone isomerase (*CHI*), flavanone 3-hydroxylase (*F3H*), flavonoid 3′-hydroxylase (*F3*′*H*), and flavonol synthase are the early biosynthetic genes, which result in the production of flavonols and different flavonoid compounds. The late biosynthetic genes include dihydroflavonol 4-reductase (*DFR*), anthocyanidin synthase (*ANS*), and UDP-glucose-flavonoid-3-*O*-glucosyltransferase (*UFGT*), which lead to the production of anthocyanins (Lepiniec et al., 2006). After glycosylation, methylation, and acylation, water-soluble anthocyanin compounds are transported to vacuoles for stable storage (Zhao and Dixon, 2010). At the transcriptional level, anthocyanin structural genes are usually conservatively regulated by the MBW protein complex containing R2R3-myeloblast (MYB), basic helix–loop–helix (bHLH), and WD40 proteins (Feller et al., 2011; Hichri et al., 2011; Xu et al., 2015). In particular, MYBs play vital roles in anthocyanin biosynthesis, and their expression levels influence anthocyanin accumulation (Chen et al., 2017; Feng et al., 2020). MYBs often interact with bHLHs to co-regulate anthocyanin biosynthesis (Patra et al., 2013; Xu et al., 2015). In the model plant *Arabidopsis thaliana*, the R2R3-MYB genes *PAP1, PAP2, MYB113*, and *MYB114*, and the bHLH genes *TT8, GL3*, and *EGL3* were identified as critical genes that participate in the regulation of anthocyanin biosynthesis (Nesi et al., 2000; Payne et al., 2000; Gonzalez et al., 2008). In *Nicotiana tabacum*, the MYB transcription factor (TF), NtAn2, can regulate anthocyanin accumulation in tobacco flowers by interacting with NtAn1a and NtAn1b to activate the promoter of *DFR* and *CHS* (Pattanaik et al., 2010; Bai et al., 2011).

Anthocyanin accumulation in leaves, usually showing a red leaf color, can greatly increase horticultural value and stress resistance. Additionally, plant materials rich in anthocyanin are important germplasm resources for genetic breeding and pigment bioengineering (Tian et al., 2015). *PdMYB118*, identified in a red leaf mutant of *Populus deltoids*, acts as a key transcriptional regulator of leaf anthocyanin accumulation (Wang et al., 2019). In peaches, a novel branch of the MYB genes, *PpMYB10.4*, can activate leaf anthocyanin accumulation and form red leaf coloration (Zhou et al., 2014). In purple kale, *BoPAP1* responds to low temperatures to induce anthocyanin biosynthesis in the leaves (Zhang et al., 2012). Similar studies have also been reported on crabapples (Tian et al., 2015), potatoes (D'Amelia et al., 2014), and *Gynura bicolor* (Shimizu et al., 2011).

*Aglaonema commutatum* is a well-known foliage plant native to tropical Asian countries such as India, Thailand, and Vietnam (Du et al., 2013). *A. commutatum* have leaves with abundant colors and mosaic points, are shade tolerant and moisture resistant, and have few pests and diseases, which makes them ideal indoor ornamental and leaf-cutting plants (Gao, 2018). Leaf color is a considerable economic trait of *A. commutatum* sought by breeders, but little is known about the molecular mechanisms of anthocyanin biosynthesis regulation in *A. commutatum*.

In this study, an R2R3-MYB and a bHLH TF were identified as potential anthocyanin biosynthesis regulators from the *A. commutatum* “Red Valentine” leaf RNA-seq database, and further analyzed. Expression trend analysis showed that the mRNA expression levels of anthocyanin structural genes correlated with those of *AcMYB1* and *AcbHLH1*. Overexpression analyses of *AcMYB1* in tobacco further demonstrated its vital role in anthocyanin regulation. Our study may lay the foundation for further genetic studies on the diversity of leaf color and can be applied to breeding plants with desirable color traits in *A. commutatum*.

## Materials and Methods

### Plant Materials and Treatment

Seedlings of *A. commutatum* “Red Valentine” that were 2-year-old were potted in the greenhouse of the South China Botanical Garden, Chinese Academy of Sciences (Guangzhou, China) and received natural light with a 60% shade cloth. The temperature and relative humidity of the greenhouse range from 15 to 34°C and 75–99%, respectively. Three stages for leaf development were defined as follows: stage 1, curly and white (S1, 7 days), stage 2, unfolded and light pink (S2, 28 days), and stage 3, mature and dark red (S3, 35 days). The leaves of three developmental stages, roots, stems, and flowers samples were frozen in liquid nitrogen immediately after collection before being stored at −80°C in November 2019 for analysis of anthocyanin content and gene expression levels.

To study the effect of light on anthocyanin biosynthesis and related gene expression, the *A. commutatum* seedlings at stage S2 were grown under 12 h light/dark cycle or dark conditions for 5 days in a phytotron (DGXM-508, Jiangnan Instrument Factory, Ningbo, China) at 28°C with 8,000 Lux light intensity. Subsequently, leaf samples were collected for analysis of gene expression and anthocyanin content. Following growth for 5 days, samples were immediately stored at −80°C for further study. Each sample contained three biological replicates.

### Anthocyanins Content Analysis

The pH difference method was used to measure the total anthocyanin content in *A. commutatum* and transgenic *Nicotiana tabacum* cv. NC89 (tobacco) tissues (Wrolstad et al., 1982). Briefly, fresh samples (0.1 g) were extracted with 2 ml methanol (with 0.05% hydrochloric acid) overnight at 0°C. The absorbance at 510 and 700 nm was measured using a microplate reader (Tecan Infinite, Männedorf, Switzerland). The following equation was used to calculate the total anthocyanin content: Δ_A_ × DF × M × 1,000/(ε × W). where Δ_A_ = (A_510_ – A_700_)pH 1.0 -(A_510_ – A_700_)pH 4.5, DF is the dilution factor, M is the molecular weight of cyanidin-3-glucoside (449.2 g mol^−1^), ε is the molar absorptivity (26,900, molar extinction coefficient (L mol^−1^· cm^−1^) for cyanidin-3-glucoside), and W is the sample weight (g).

### Transcript Isolation and Sequence Analysis

The leaf transcriptome data of *A. commutatum* (Accession number: PRJNA793608) were used to screen for R2R3-MYB-like and bHLH-like genes by BLASTx searching from Nr and Swiss-Prot protein databases based on (i) similarity scores with the known anthocyanin-related TF genes in other species and (ii) correlations with anthocyanin structural gene expression. The transcripts were obtained from transcriptome assembly data, and the coding sequences were cloned using the SMARTer RACE cDNA Amplification Kit (Takara Biomedical Technology Co., Ltd.; Beijing, China), and amplified *via* PCR with 2× Super Pfx DNA Polymerase (Cowin Biotech Co., Ltd.; Taizhou, China) and the pClone007 Versatile Simple Vector Kit (TSINGKE Biotech Co., Ltd.; Beijing, China) for Sanger sequencing and subsequent analyses. DNAMAN (v8.0.8.789) was used to analyze the amino acid sequence alignment and MEGA (v7.0.26) was used to construct phylogenetic trees using the neighbor-joining method with 1,000 bootstrap replicates. The GenBank accession numbers of the MYB and bHLH proteins are listed in Supplementary Table S1.

### RNA Extraction, cDNA Synthesis, and Gene Expression Analysis

Total RNA was isolated from tobacco as well as the roots, stems, flowers, and leaves of *A. commutatum* using a plant RNA kit (Polysaccharides and Polyphenolics-rich, Hua Yueyang, Beijing, China) with RNase-free DNase I (Takara Biomedical Technology Co., Ltd.; Beijing, China) to remove genomic DNA contamination. RNA (1 μg) was used for cDNA synthesis using the TransScript II One-Step gDNA Removal and cDNA Synthesis kit (TransGen Biotech Co., Ltd.; Beijing, China).

Key genes related to anthocyanin biosynthesis were screened from the *A. commutatum* “Red Valentine” leaf RNA-Seq database (BioProject ID: PRJNA793608). We identified 10 anthocyanin biosynthetic genes: *AcCHS, AcCHS2, AcCHI, AcF3H, AcF3'H, AcDFR1, AcDFR3, AcANS, AcUFGT1*, and *AcUFGT2*. The expression levels of *AcMYB1* and *AcbHLH1* as well as these 10 genes were analyzed in the root, stem, flower, and three developmental leaf stages of *A. commutatum*. The expression levels of *AcMYB1* in transgenic tobacco leaves and flowers were also determined. The expression levels of nine structural genes and two bHLH TF genes (*NtPAL, NtCHS, NtCHI, NtF3H, NtF3'H, NtF3'5'H, NtIDFR, NtANS, NtUFGT, NtAn1a*, and *NtAn1b*, respectively) involved in anthocyanin biosynthesis were compared in the transgenic and control tobacco leaves and corollas. Finally, the mRNA abundance of six anthocyanin structural genes (*NbCHS, NbCHI, NbF3H, NbDFR, NbANS, NbUFGT*) in *Nicotiana benthamiana* leaves were examined 8 days after infiltration. Gene expression levels were determined using real-time quantitative PCR (qRT-PCR) with PerfectStart Green qPCR SuperMix (TransGen Biotech Co., Ltd., Beijing, China) with a LightCycler 480 II (Roche, Mannheim, Germany) according to the following conditions: 94°C for 30 s, 45 cycles at 94°C for 5 s, 60°C for 30 s. Elongation factor 1-alpha (*AcEF-1*α, Accession number: OM688333), *NtActin* (Accession number: X69885), and *NbActin* (Accession number: JQ256516) were used as reference genes. The Ct value of each sample was calculated *via* LightCycler 480 software (Roche, version: 1.5.1.62) and the relative expression was determined using the 2^−Δ*ΔCT*^ method (Livak and Schmittgen, 2001), where ΔCt = Ct (target gene)–Ct (reference gene) and ΔΔCT = ΔCt (experimental group)–ΔCt (control group). Gene-specific primer pairs are listed in Supplementary Table S2. Three biological replicates and three technical replicates were performed.

### Subcellular Localization

The *AcMYB1* and *AcbHLH1* open reading frames, without the stop codons, were inserted into the BglII and KpnI sites of the pSAT6-EYFP-N1vector which is a yellow fluorescent protein (YFP) driven by the 35S Cauliflower mosaic virus (35S) promoter. The final plasmids, 35S::AcMYB1-YFP, 35S::AcbHLH1-YFP, control 35S::YFP, and nuclear marker 35S::mCherry, were introduced into *A. thaliana* mesophyll protoplasts by polyethylene glycol (PEG)-mediated transient transformation (Yoo et al., 2007). Co-transformation with the control 35S::YFP and nuclear marker 35S:: mCherry were used as a negative control. After incubation at 20°C for 20 h, the protoplasts were detected using a Zeiss LSM 510 confocal microscope (Zeiss, Jena, Germany).

### Yeast Two-Hybrid Assay

Yeast two-hybrid (Y2H) analysis was performed using the Matchmaker Two-Hybrid System 3 (Clontech; Takara Bio USA, Inc.; San Jose, CA, United States). The coding region of *AcbHLH1* was inserted into the bait vector pGBK-T7 (GAL4 DNA-binding domain), and *AcMYB1* was fused to the prey vector pGAD-T7 (GAL4 activation domain). Vector pGADT7-T and pGBKT7-53 were used as the positive controls. All constructs were transformed into the yeast strain AH109 (Clontech, Takara Bio USA, Inc.; San Jose, CA, United States) using the PEG/LiAC method, according to the protocol handbook. All transformed yeast cells were selected on a synthetic drop-out medium without leucine and tryptophan (SD–Leu–Trp) at 30°C for 3 days. Colonies that survived from double selection plates were then screened for growth on a quadruple selection SD medium lacking adenine, histidine, leucine, and tryptophan (SD-Ade-His-Leu-Trp) containing 30 mM 3-amino-1,2,4-triazole (3-AT) solution and 25 mg/L X-α-Gal.

### Bimolecular Fluorescence Complementation

For the biomolecular fluorescence complementation (BiFC) assay, the *AcMYB1* and *AcbHLH1* ORFs, without stop codons, were inserted into the pSPYNE and pSPYCE vectors containing the N- and C-terminal halves of YFP (Walter et al., 2004). Recombinant plasmids were then introduced into *A. thaliana* mesophyll protoplasts by PEG-mediated transient transformation (Waadt et al., 2008). After incubation at 20°C for 20 h, the protoplasts were observed using a Zeiss LSM 510 confocal microscope (Zeiss, Jena, Germany).

### Overexpression of *AcMYB1* in Tobacco

To overexpress *AcMYB1* in tobacco, the coding sequence of *AcMYB1* was inserted into the pGreen-C17 vector, which was triggered under the control of the CaMV 35S promoter. The resulting vector, pGreen-C17-AcMYB1, was transferred into *Agrobacterium tumefaciens* strain EHA105 and then transformed into tobacco *via* the leaf disc method (Zhang et al., 2007). Empty vector-infected tobacco plants were used as controls. The T1-generation leaves and corollas from the three transgenic lines and control plants were collected for the anthocyanin concentration (Section Anthocyanins Content Analysis) and gene expression (Section RNA Extraction, cDNA Synthesis, and Gene Expression Analysis) analyses.

### Transient Expression Assay

The transient over-expression experiments in *N. benthamiana* leaves were performed according to the method described by Palapol et al. (2009). Briefly, 35S-promoter-driven AcMYB1 and AcbHLH1 constructs were inserted into *Agrobacterium tumefaciens* strain GV3101 *via* electroporation. *N. benthamiana* plants with 6–8 leaves were solely or simultaneously infiltrated with a needleless 1-ml syringe into the abaxial sides of the fourth or fifth leaves, and Agrobacteria with an empty vector was used as a control. Anthocyanin content was measured and digital photographs were taken 8 days after infiltration. Anthocyanin content was measured with the pH difference method as described in Section Anthocyanins Content Analysis.

### Statistical Analysis

All experiments were performed on at least three independently grown biological replicates. All values represent the mean ± SE. Differences between the treatment groups were examined using SPSS software (one-way ANOVA, Duncan test), and the significant differences (*p* < 0.05) are indicated with different letters, respectively.

## Results

### Determination of Anthocyanin Concentration in *A. commutatum*

**Figure 1:**
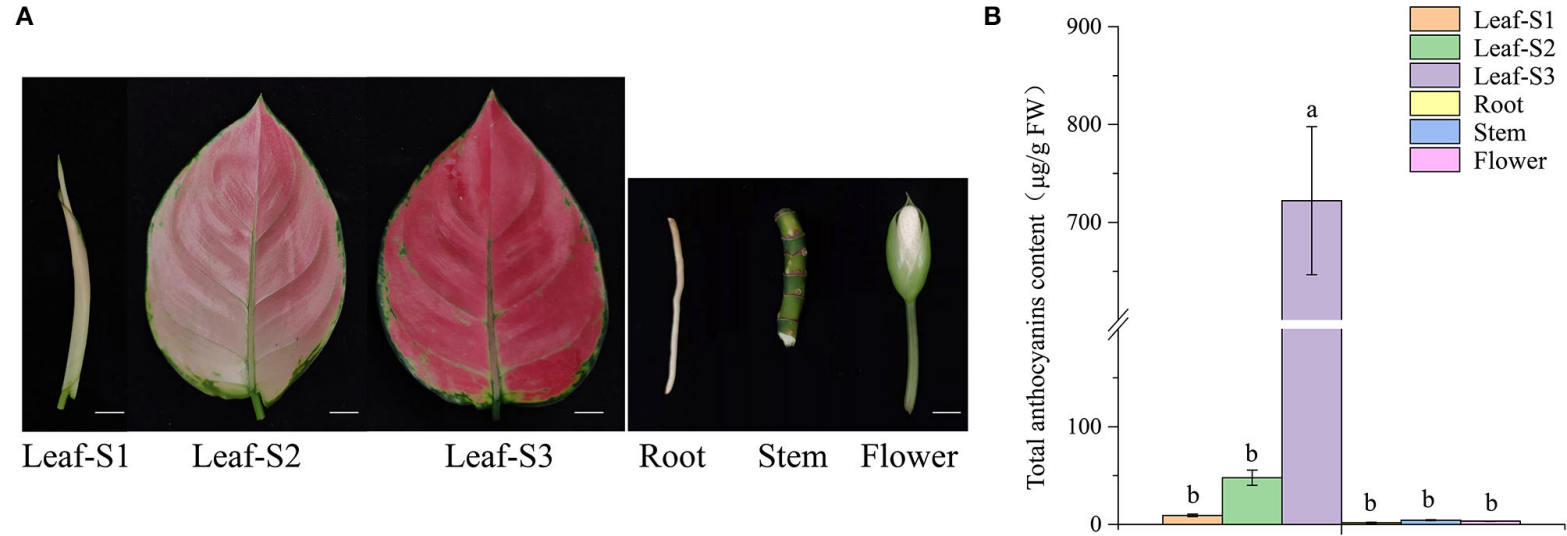
Anthocyanin content in the leaves, at the three developmental stages; roots; stems; and flowers of *Aglaonema commutatum* “Red Valentine.” **(A)** The phenotypes of the leaves at the three developmental stages, S1, S2, and S3; roots; stems; and flowers. Bar: 1 cm. **(B)** Anthocyanin content in the different tissues.

Leaves of *A. commutatum* “Red Valentine” has white color and stay curly at leaf-S1. At leaf-S2, leaf color changes from white to light pink and appears deep red at leaf-S3 (Figure 1A). Anthocyanin content in the leaves of *A. commutatum* significantly increased with developmental age. Additionally, trace amounts or no anthocyanins were detected in the roots, stems, and flowers (Figure 1B).

### Isolation and Sequence Analysis of *AcMYB1* and *AcbHLH1*

**Figure 2:**
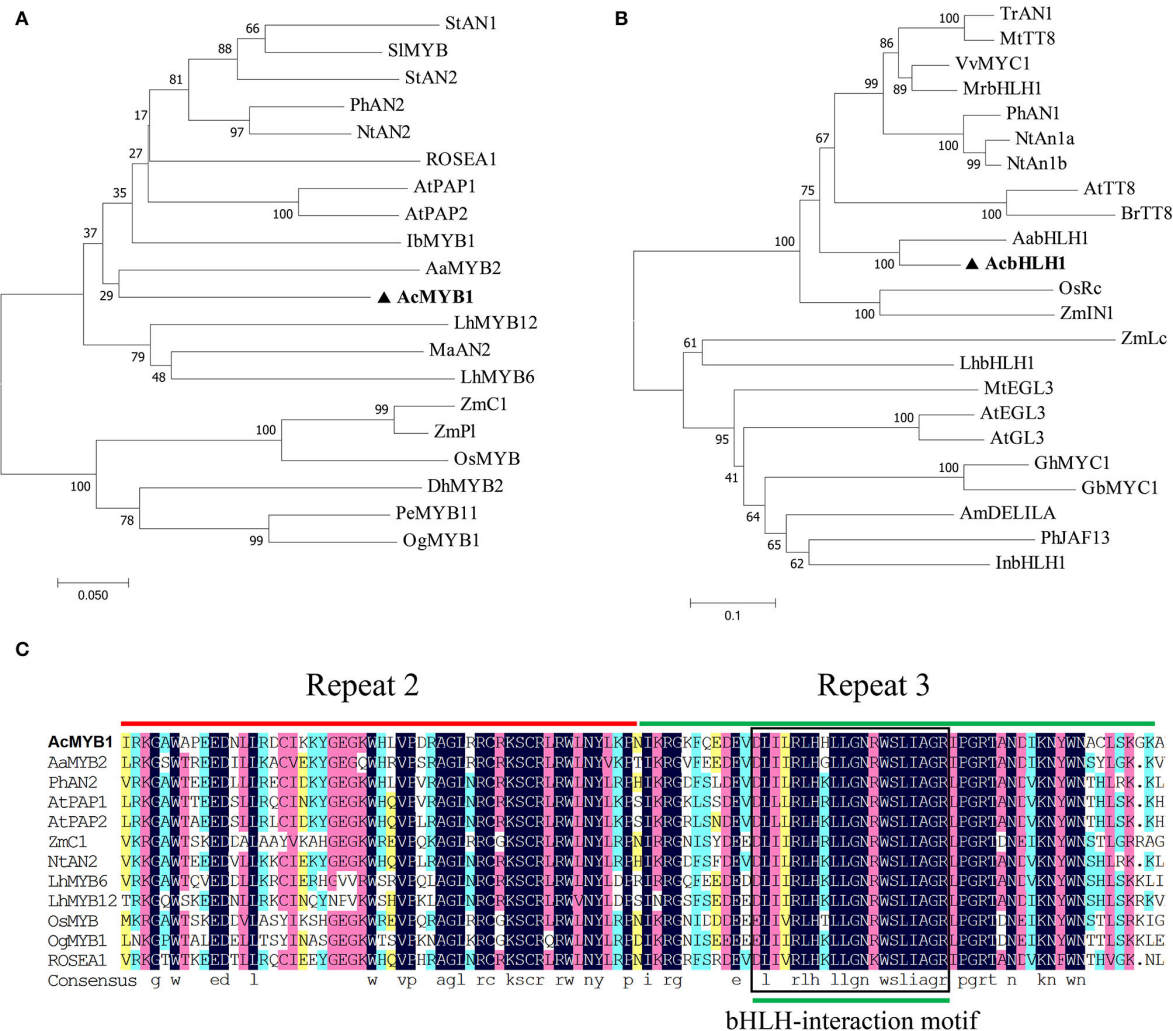
Phylogenic and sequence alignment analyses of *A. commutatum* “Red Valentine” AcMYB1 and AcbHLH1. Phylogenetic relationships of **(A)** AcMYB1 and **(B)** AcbHLH1 with known anthocyanin MYB and bHLH transcription factors, respectively, from monocotyledon and other species. The neighbor-joining method with 1,000 bootstrap replications was performed using MEGA (v7.0.26). **(C)** Amino acid sequence alignments of the R2 and R3 domains in AcMYB with the functionally characterized R2R3-MYBs regulator using DNAMAN (v8.0.8.789). The R2 and R3 domains are shown with red and green lines, respectively, and the bHLH-interacting motif is indicated in the black box. bHLH; basic helix–loop–helix, MYB: myeloblast. The GenBank accession numbers of the MYB and bHLH proteins are listed in Supplementary Table S1.

In this study, R2R3-MYB and bHLH TFs were isolated from the leaf transcriptome database of *A. commutatum* “Red Valentine.” The candidate genes were cloned from the cDNA of *A. commutatum* leaves by RACE PCR, sequenced, and named *AcMYB1* and *AcbHLH1*. The results showed that *AcMYB1* and *AcbHLH1* contained 969 and 2,115 bp ORFs, encoding proteins of 322 and 712 amino acids, respectively. The neighbor-joining phylogenetic tree showed that AcMYB1 clustered with monocot-positive anthocyanin regulators, including AaMYB2, LhMYB6, LhMYB12, and MaAN2 (Figure 2A). Similarly, AcbHLH1 formed a cluster with AabHLH1 and other bHLH TFs associated with anthocyanin biosynthesis in several plant species (Figure 2B). Alignment analysis showed that a highly conserved R2R3 domain is contained in the AcMYB1 protein for DNA binding at the N-terminus, which contains a crucial bHLH-interacting motif in the R3 domain for interactions with bHLH TFs (Figure 2C) (Zimmermann et al., 2004). We also found an N-terminal MYB-interacting region and conserved bHLH domain in the AcbHLH1 protein (Supplementary Figure S1) (Pattanaik et al., 2008). These results suggested that AcMYB1 and AcbHLH1 may interact and play roles in anthocyanin biosynthesis.

### Expression Levels of *AcMYB1, AcbHLH1*, and Anthocyanin Structural Genes

**Figure 3:**
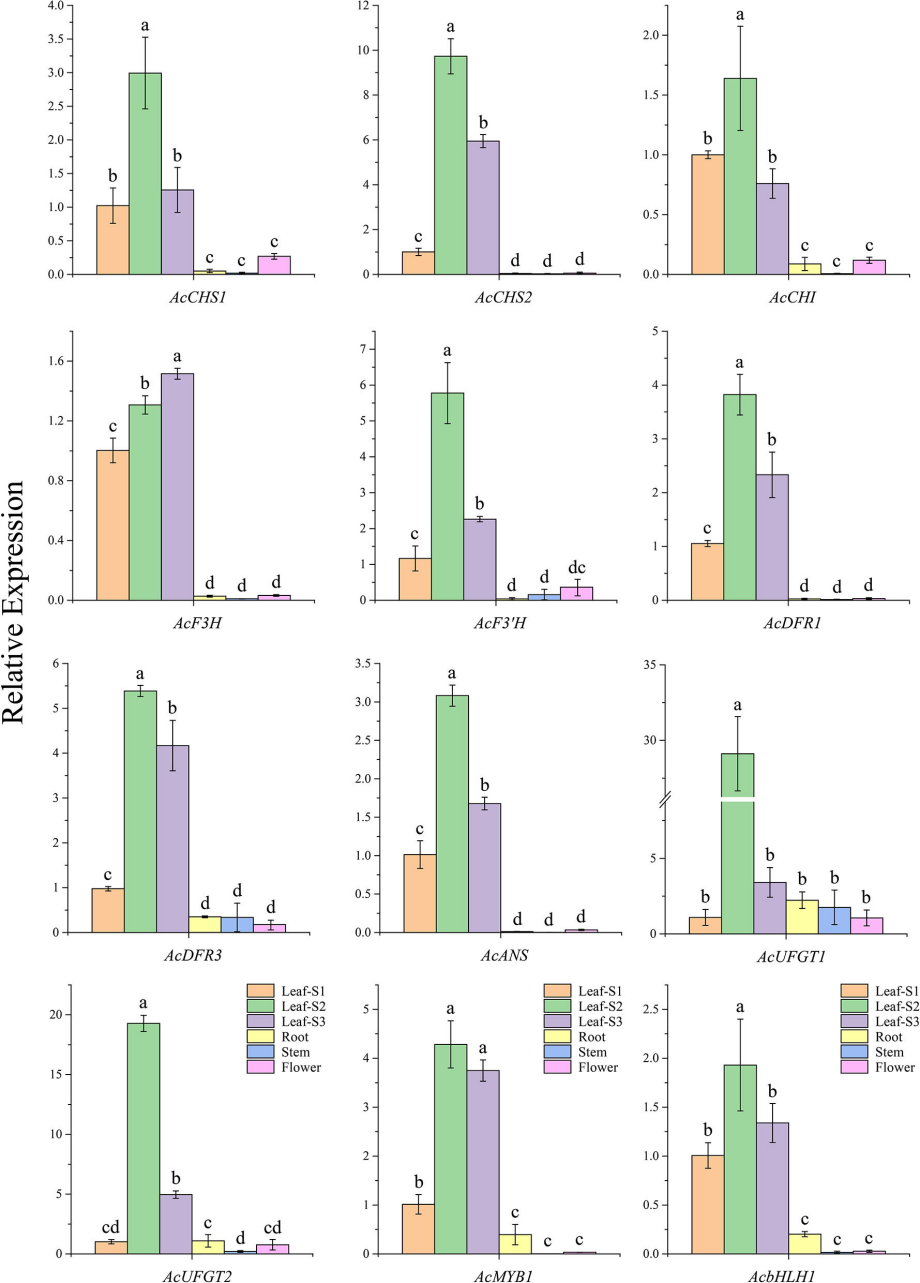
The relative expression levels of 10 anthocyanin structural genes, *AcMYB1*, and A*cbHLH1* in various tissues of *A. commutatum* “Red Valentine” by qRT-PCR. The relative expression was calculated based on the 2^−Δ*ΔCT*^ method using AcEF-1α gene as the reference gene.

The gene expression analyses showed that all the candidate structural genes, including *AcMYB1* and *AcbHLH1*, had significantly higher expression levels in the three developmental leaf stages compared to those in the other tissues and reached a peak at leaf stage two except *AcF3H* (Figure 3). Furthermore, the correlation analysis revealed that the gene expression correlation coefficient between AcMYB1 or AcbHLH1 and structural genes was in the range of 0.73–0.99 and 0.75–0.98. This result implies that transcript abundance of *AcMYB1* and *AcbHLH1* are correlated with those of the most anthocyanin structural genes and they are likely involved in the regulation of anthocyanin biosynthesis in *A. commutatum*.

### Localization and Protein–Protein Interaction of *AcMYB1* and *AcbHLH1*

**Figure 4:**
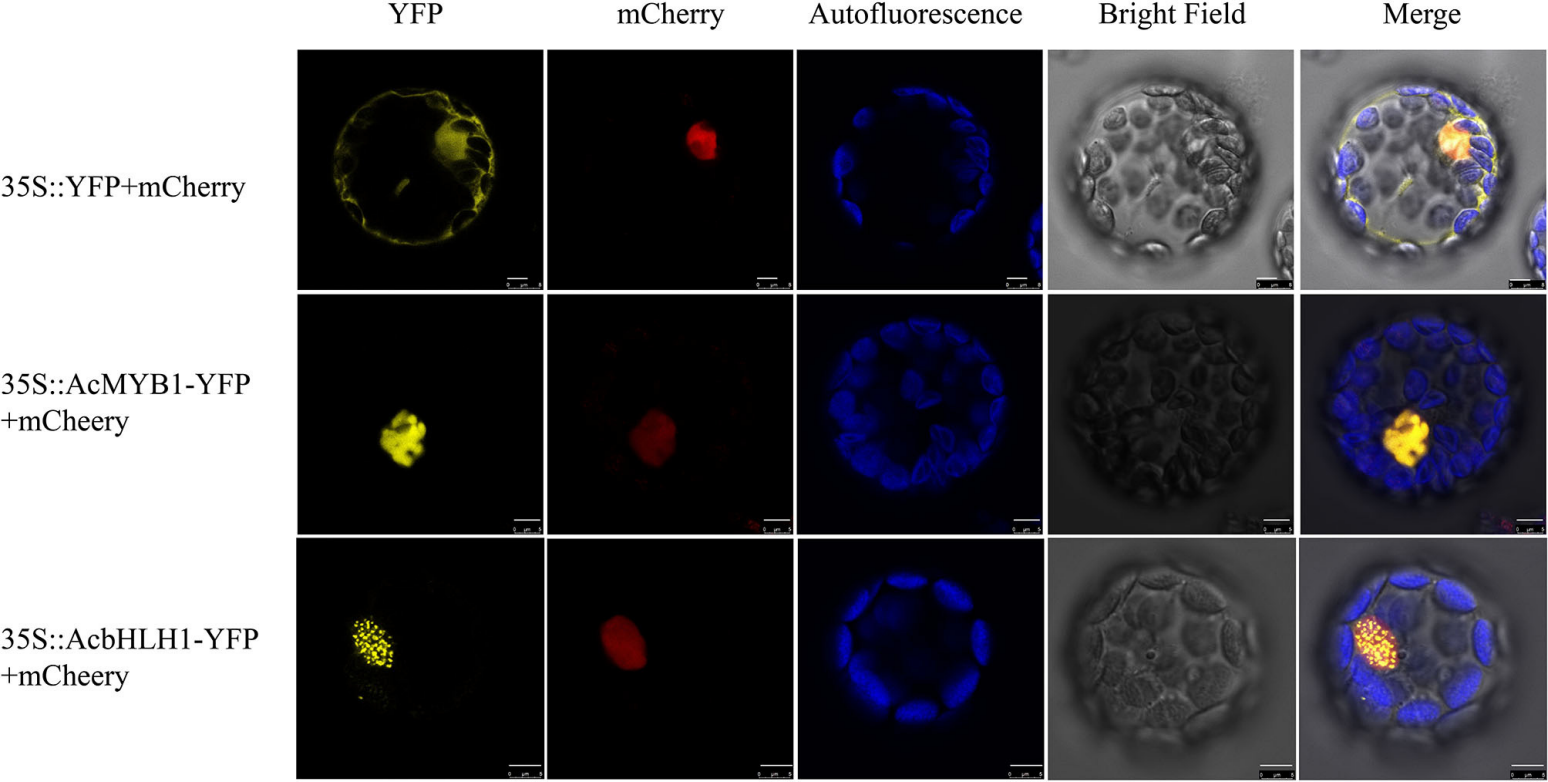
Subcellular localization of AcMYB1 and AcbHLH1 proteins in *Arabidopsis thaliana* mesophyll protoplasts. Transient expression of the 35S::YFP-mCherry control, 35S::AcMYB1-YFP, and 35S::AcbHLH1-YFP, showing YFP fluorescence, mCherry nuclear localization, chlorophyll autofluorescence, bright field, and merged images. Scale bar: 5 μm. YFP, yellow fluorescence protein.

**Figure 5:**
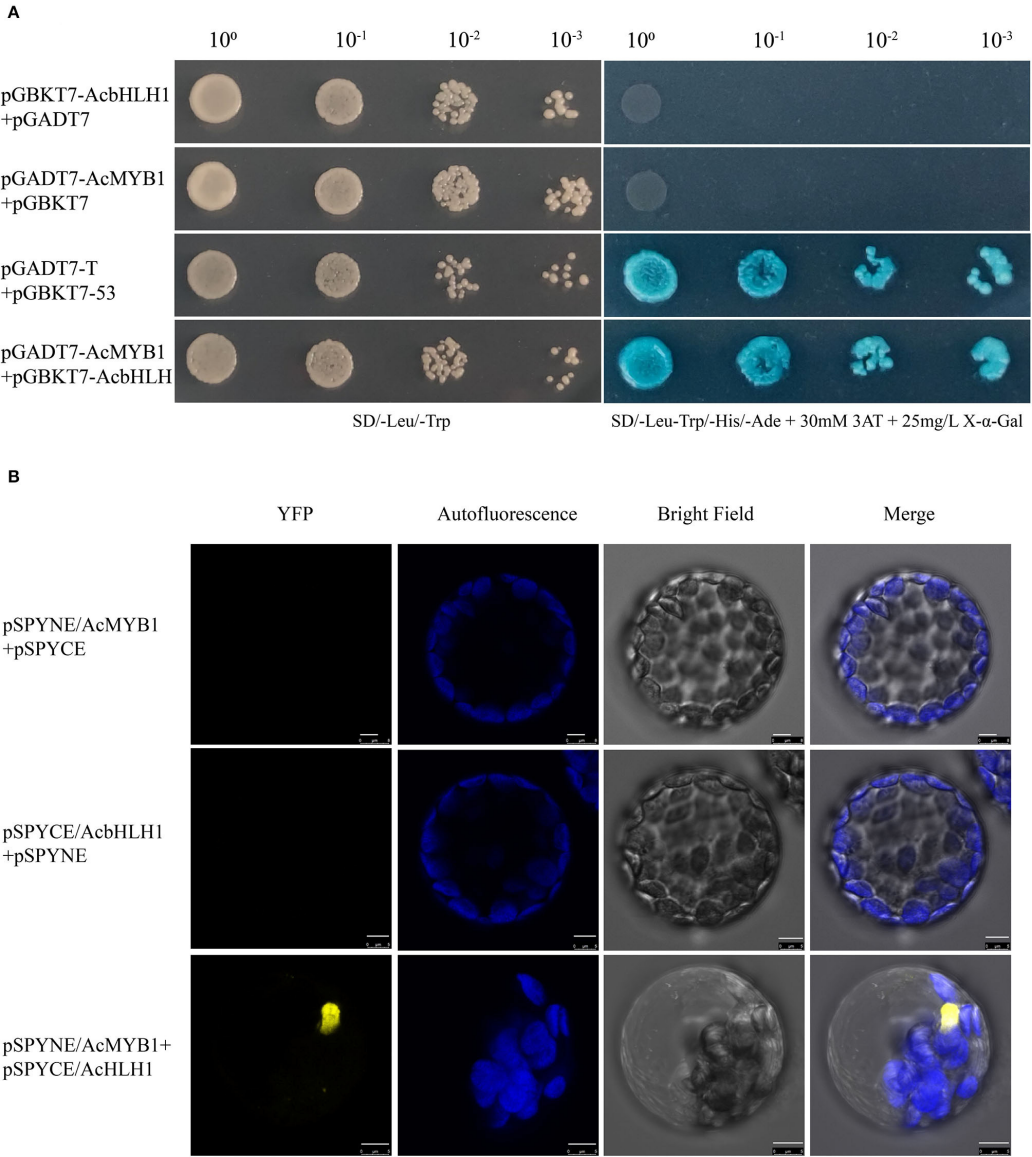
Interactions between AcMYB1 and AcbHLH1 determined by Y2H and BiFC assays. **(A)** Y2H assay of AcMYB1 with AcbHLH1: co-transformed colony, pGADT7-AcMYB1 + pGBKT7-AcbHLH1; positive control, pGADT7-T + pGBKT7-53. **(B)** BiFC assay showing the fluorescence signal of co-expressed pSPYNE/AcMYB1 + pSPYCE/AcbHLH1 in *Arabidopsis thaliana* leaf protoplasts. Representative images under the YFP fluorescence, chlorophyll autofluorescence, bright field, and merged images. Scale bar: 5 μm.

Subcellular localization analysis in *A. thaliana* leaf protoplasts revealed that both AcMYB1 and AcbHLH1 were specifically localized in the cell nucleus (Figure 4). In *A. commutatum*, AcMYB1 and AcbHLH1 showed conserved interacting motifs, similar mRNA expression patterns in different tissues, and co-localization in the nucleus, suggesting a possible interaction. To test this hypothesis, we used Y2H and BiFC assays. For the Y2H assay, all the transformed colonies grew well on SD/-Leu/-Trp, indicating their successful transformation. The co-transformed colonies of pGADT7-AcMYB1 + pGBKT7-AcbHLH1 displayed distinct blue coloration on SD/-Leu/-Trp/-His/-Ade, indicating that AcMYB1 and AcbHLH1 physically interacted (Figure 5A). In the BiFC assays, YFP fluorescence signals were observed only when pSPYNE/AcMYB1 and pSPYCE/AcHLH1 were co-expressed and no fluorescence was detected in cells that contained control vectors (Figure 5B). These results further confirm that AcbHLH1 and AcbMYB1 may interact in *A. commutatum* cells.

### Functional Analysis of *AcMYB1* in Tobacco

**Figure 6:**
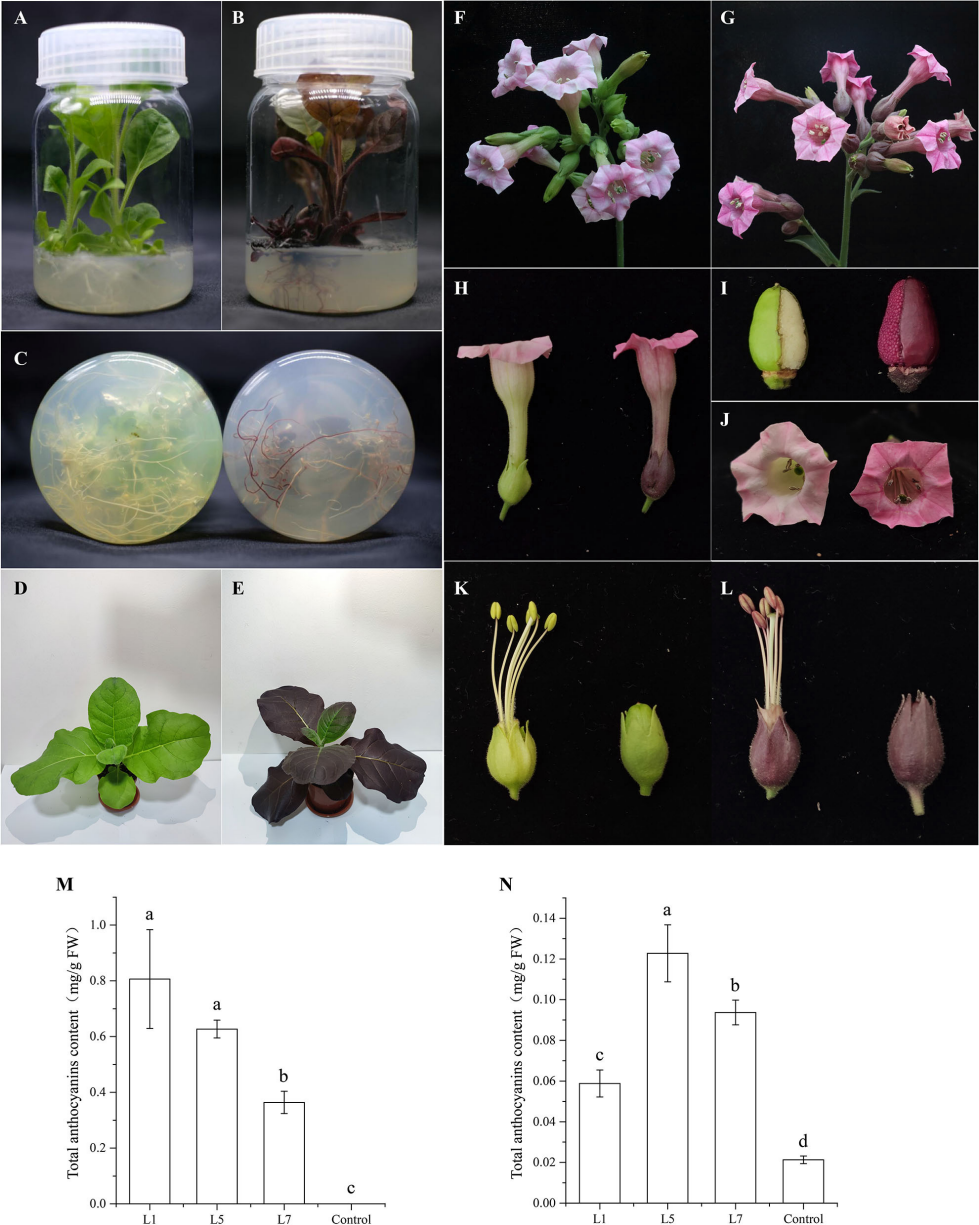
Phenotypes and anthocyanin content of transgenic *Nicotiana tabacum* cv. NC89 plants overexpressing (OE)-AcMYB1 and empty vector controls. Culture seedling phenotypes of **(A)** control and **(B)** OE-AcMYB1 lines. **(C)** Root phenotypes of control (left) and OE-AcMYB1 (right) lines. Seedling phenotypes of **(D)** control and **(E)** OE-AcMYB1 lines. Flower phenotypes of **(F)** control and **(G)** OE-AcMYB1 lines. **(H)** corolla, **(I)** ovary wall, and **(J)** seed coat phenotypes of control (left) and OE-AcMYB1 (right) lines. Anther, filaments, and calyx phenotypes of **(K)** control and **(L)** OE-AcMYB1 lines. Anthocyanin content of leaves **(M)** and corollas **(N)** in OE-AcMYB1 lines L1, L5, and L7 and a control.

To investigate the role of *AcMYB1* in regulating the anthocyanin biosynthetic pathway, overexpression (OE) under the control of the CaMV-35S promoter was carried out in tobacco. More than 20 independent transgenic lines were generated using genomic PCR. The results of phenotypic changes showed that pigment levels were significantly increased in both vegetative and reproductive tissues relative to that in the control plants. In particular, the OE-AcMYB1 line showed a remarkable change in leaf color, and the mature leaves of the transgenic plants displayed an observably darker red color than that of the control plants. In addition, the corolla color of OE-AcMYB1 tobacco changed from light pink to deep red, and increased anthocyanin accumulation was markedly visible in the anthers, filaments, calyxes, ovary walls, and seed coats (Figures 6A–L). Anthocyanin content determination confirmed that the anthocyanin extracted from the three lines of OE-AcMYB1 tobacco leaves and corollas were markedly higher than those in the controls (Figures 6M,N).

**Figure 7:**
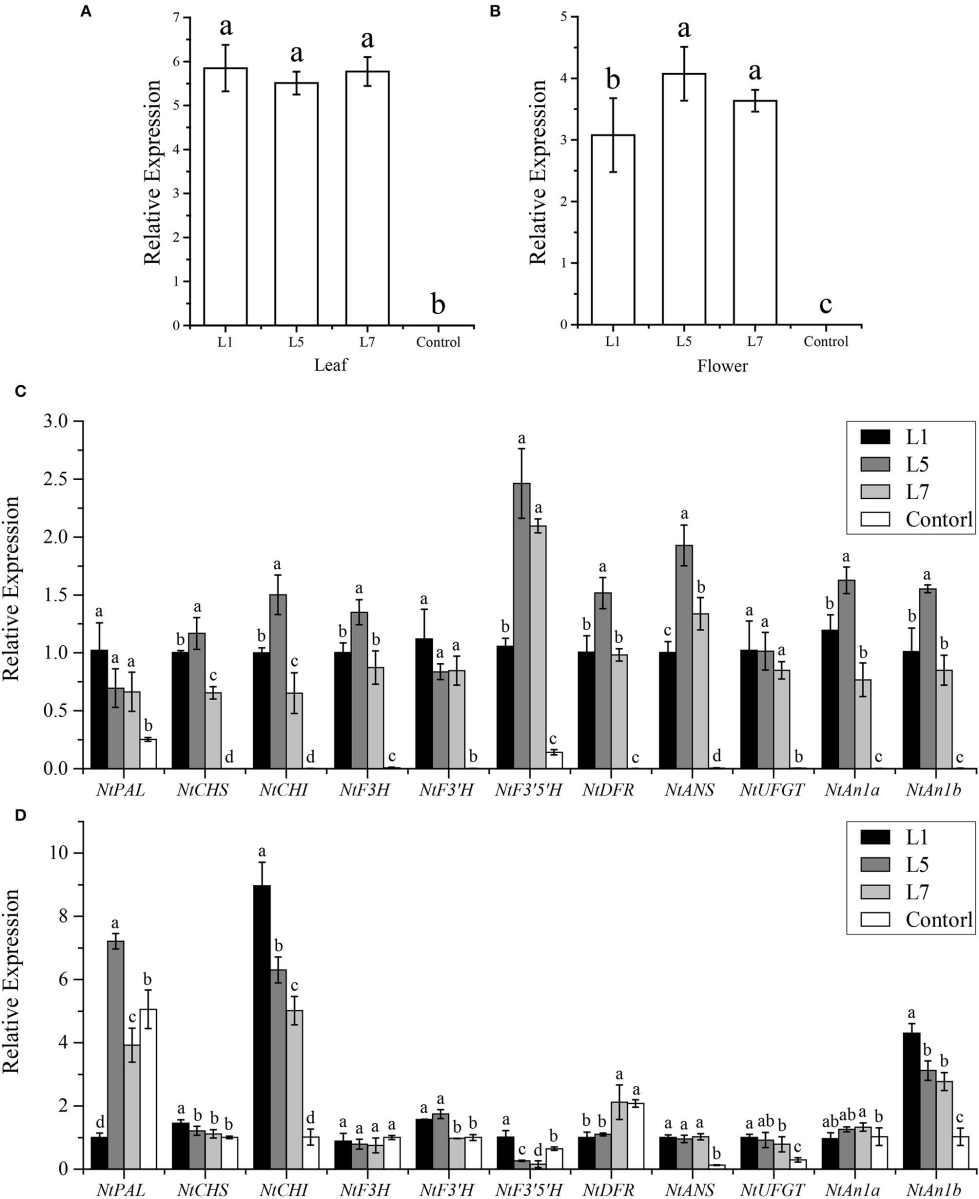
Expression analysis of anthocyanin biosynthesis pathway genes in the leaves and corollas of *Nicotiana tabacum* cv. NC89 using qRT-PCR. The expression patterns of *AcMYB1* in the **(A)** leaves and **(B)** corollas. Relative expression levels of anthocyanin pathway-related genes in the leaves **(C)** and corollas **(D)**. L1, L5, and L7 represent the three transgenic tobacco lines. The relative expression was calculated based on the 2^−Δ*ΔCT*^ method using the *NtActin* gene as the reference gene.

To reveal the potential target genes in OE-AcMYB1 transgenic plants, the mRNA expression level of structural genes and two bHLH TF genes involved in anthocyanin biosynthesis were verified by RT-PCR assay in tobacco leaves and corollas. A high expression level of AcMYB1 was first confirmed in three lines by qRT-PCR (Figures 7A,B). Expression analysis showed that the 11 anthocyanin regulatory genes were significantly upregulated in the leaves of all three transgenic lines compared to those of the control plants (Figure 7C). However, in the corollas of the three transgenic tobacco plants, only *NtCHI, NtANS, NtUFGT*, and *NtAn1b* showed significantly higher expression levels than that of the control (Figure 7D). These results indicated that AcMYB1 could upregulate or activate the expression of key anthocyanin structural genes and bHLH TFs, ultimately promoting anthocyanin accumulation in transgenic tobacco.

### Transient Expression of *AcMYB1* and *AcbHLH1* in *N. benthamiana* Leaves

**Figure 8:**
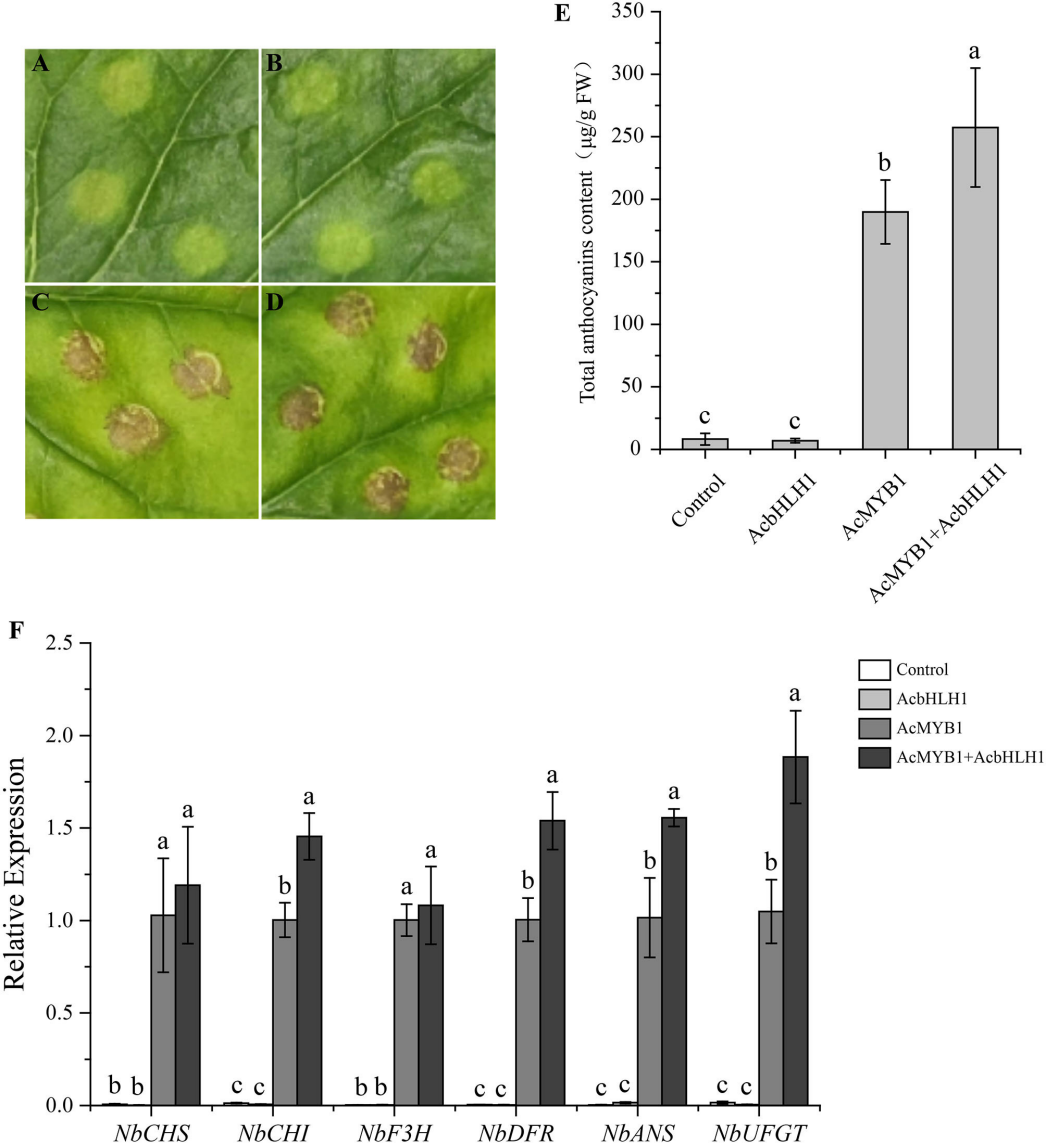
Transient over-expression assay in *N. benthamiana* leaves. Images of infiltration sites 8 days after transformation of control vector **(A)**, AcbHLH1 **(B)**, AcMYB1 **(C)**, and AcMYB1+AcbHLH1 **(D)**. **(E)** The anthocyanin content of *N. benthamiana* leaves after infiltration. **(F)** The expression analysis of six anthocyanin biosynthetic genes of *N. benthamiana* after infiltration. The relative expression was calculated based on the 2^−Δ*ΔCT*^ method using the *NbActin* gene as the reference gene.

An Agrobacterium-mediated transient assay was further performed to investigate the regulation of AcMYB1 and AcbHLH1 on anthocyanin biosynthesis. The results showed that a slight accumulation of anthocyanin was detected in the leaves inoculated with the control or AcbHLH1 construct, while patches of anthocyanin were observed in AcMYB1 and AcMYB1 + AcbHLH1 inoculated leaves (Figures 8A–E). The anthocyanin content in AcMYB1 + AcbHLH1 leaves is 1.36 times that of the AcMYB1 leaves (Figure 8E). Moreover, the expression of anthocyanin biosynthetic genes were strongly up-regulated in the leaves infiltrated with AcMYB1 and AcMYB1 + AcbHLH1 constructs (Figure 8F). Interestingly, *NbCHI, NbANS, NbDFR*, and *NbUFGT* displayed significantly higher expression levels in simultaneous inoculation of 35S::*AcMYB1* and 35S::*AcbHLH1* constructs than solely infiltrating with 35S::*AcMYB1*. Taken together, we predicted that the ability of AcMYB1 in anthocyanin regulation could enhance by the interaction with AcbHLH1.

### Influence of Light on Gene Expression and Anthocyanin Accumulation in *A. commutatum*

**Figure 9:**
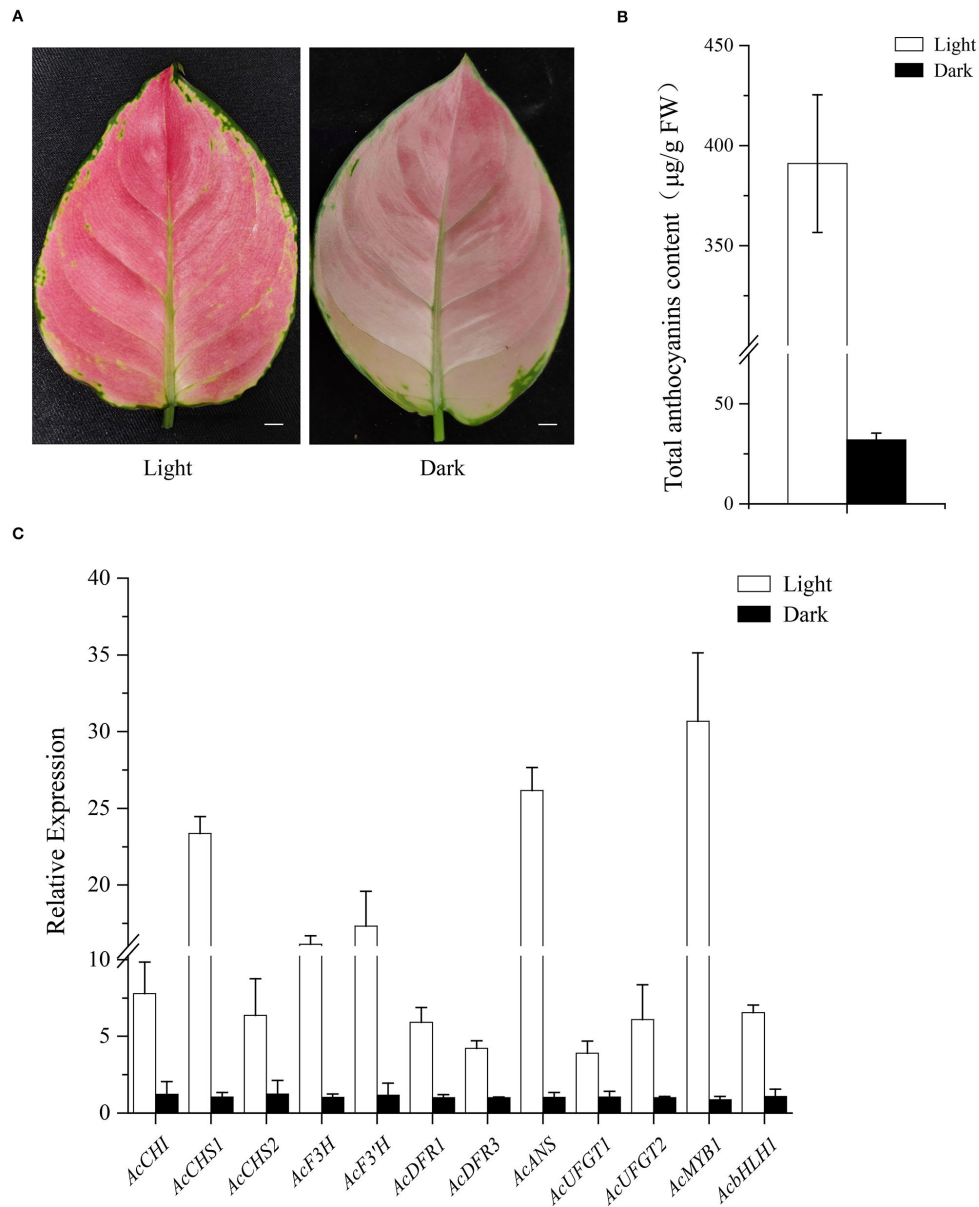
Influence of light on leaf color. **(A)** Leaf phenotype after 5 days light (left) or dark (right) treatment. **(B)**. Anthocyanin content of light and dark treatment. **(C)** Expression analysis of *AcMYB*1, *AcbHLH1*, and 10 structural genes of the anthocyanin pathway was determined by qRT-PCR assay with AcEF-1α as a reference gene. Bar: 1 cm.

Comparative analysis was conducted between 2-year-old *A. commutatum* “Red Valentine” seedlings after growing in the dark or light for 5 days. As shown in Figure 9A, the leaf color was bright red in light treatment plants and pale pink in dark-grown plants. The leaf anthocyanin content in light treatment seedlings is 11.2-fold higher than that of the seedlings growing in dark (Figure 9B). Furthermore, the transcriptional level of AcMYB1, AcbHLH1, and 10 anthocyanin structural genes in light-grown conditions were all noticeably higher in comparison with the seedlings growing in dark (Figure 9C). The results reveal that light can strongly induce the expression of anthocyanin-related genes and promote the anthocyanin accumulation in *A. commutatum* “Red Valentine” leaf.

## Discussion

Recently, more attention has been given to the cultivation of ornamental plants with colored leaves; a phenotype that is mainly obtained by the accumulation of anthocyanins (Lightbourn et al., 2008; Li et al., 2021; Huang et al., 2022). Multiple R2R3-MYB and bHLH TFs have been reported as key regulators of anthocyanin biosynthesis (Allan et al., 2008; Petroni and Tonelli, 2011; Qin et al., 2022). *A. commutatum* is an excellent foliage plant with abundant leaf coloration; therefore, acquiring increasing importance for breeding desirable leaf color traits. However, few studies have focused on the molecular mechanisms underlying anthocyanin biosynthesis and regulation in *A. commutatum*.

Many R2R3-MYBs involved in the regulation of anthocyanin biosynthesis have been identified at the genetic and molecular levels (Nuraini et al., 2020; Yin et al., 2021), such as PeMYB2, PeMYB11, and PeMYB12 in *Phalaenopsis* spp. (Hsu et al., 2015); MaAN2 and MaMYBA in *Muscari armeniacum* (Chen et al., 2017, 2019); MdMYB10 and MdMYB6 in *Malus* × *domestica* (Espley et al., 2009; Xu et al., 2020); and FhPAP1 from *Freesia hybrida* (Li et al., 2020). In this study, AcMYB1 had the typical characteristics of an R2R3-MYB transcription factor. The AcMYB1 protein contains a highly conserved R2 and R3 repeat in the N-terminal region and a bHLH- interacting motif in the R3 domain. Phylogenetic analysis indicated that AcMYB1 was grouped into the AN2 subgroup, which is represented by PhAN2 and AtPAP1 (Allan et al., 2008). Besides, AcMYB1 was closest to the AaMYB2 transcription factor. AaMYB2 was reported to be a key regulator of anthocyanin biosynthesis in the spathes and leaves of *Anthurium andraeanum*, another member of the Araceae (Li et al., 2016). Moreover, based on the expression patterns, the anthocyanin biosynthetic genes, except for AcF3H, in *A. commutatum* share similar trends with those of *AcMYB1* in the different tissues. These results suggest a potential role for AcMYB1 in regulating anthocyanin accumulation in *A. commutatum*.

The enzymes of anthocyanin structural genes are mainly regulated at the transcriptional level by the interaction between R2R3-MYB and bHLH transcription factors (Koes et al., 2005; Xu et al., 2014). Examples include the ZmC–ZmLc (R2R3-MYB–bHLH) complex in *Zea mays* (Dooner et al., 1991), PhAN2–PhAN1 complex in *Petunia* spp. (Spelt et al., 2000), and NtAn2–NtAn1 complex in *N. tabacum* (Bai et al., 2011). In this study, AcMYB1 formed a heterodimer with AcbHLH1 and could play a key role in the regulation of leaf color in *A. commutatum* (Figure 5). Additionally, in the three lines of OE-AcMYB1 tobacco leaves, the expression levels of both NtAn1a and NtAn1b were significantly upregulated (Figure 7C), suggesting that AcMYB1 may activate anthocyanin-related bHLH in tobacco.

Heterologous expression in model plants can quickly provide the basis for functional identification of target genes. Tobacco is one of the most widely studied model plants in verifying the functions of anthocyanin regulators (Vimolmangkang et al., 2013; Huang et al., 2016; Zhao et al., 2022). For example, HtMYB2 (Gao et al., 2020), EsMYB9 (Huang et al., 2017), AaMYB2 (Li et al., 2016), and IbMYB1a (An et al., 2015) were found to regulate tobacco anthocyanin biosynthesis to varying degrees. Similarly, in this study, overexpression of AcMYB1 in tobacco displayed striking changes in anthocyanin accumulation in both vegetative and reproductive tissues at various developmental stages (Figures 6A–L). A remarkable increase in anthocyanin was also observed in OE-AcMYB1 lines (Figures 6M,N). We further investigated the function of AcMYB1 in regulating anthocyanin biosynthesis by examining the expression patterns of anthocyanin biosynthesis pathway-related genes in tobacco. In the leaves of the three OE-AcMYB1 lines, all anthocyanin structural genes and endogenous bHLH genes were highly expressed, whereas little or no mRNA abundance was detected in control tobacco. In the corolla, the activation of *AcMYB1* was not as strong as that of leaves; only *NtCHI, NtANS, NtUFGT*, and *NtAn1b* were significantly upregulated in the three OE-AcMYB1 lines. Overall, the heterogeneous expression analysis strongly supports the notion that AcMYB1 plays an important role in determining red leaf coloration in *A. commutatum*.

It has been reported in a variety of plants that strong light can increase the expression of anthocyanin-related genes and promote the accumulation of anthocyanins, while under dark or weak light conditions, the expression and the biosynthesis of anthocyanins were both inhibited, usually showing white color or pale phenotype (Cominelli et al., 2008; Azuma et al., 2012; Zhang et al., 2018). After the dark treatment for 6 days, the color of the lily flower became lighter with a decrease of anthocyanin content, and the expression of the structural gene *LhDFR* and the regulatory gene *LhbHLH2* was only one-fifth of that under light conditions, while another anthocyanin-related gene *LhbHLH1* was not affected by shading (Nakatsuka et al., 2009). Similarly, the anthocyanin content and the expression abundance of anthocyanin genes, including *MrF3H, MrF3'H, MrDFR, MrANS, MrUFGT*, and *MrMYB1* of the Chinese bayberry fruit were significantly inhibited after bagging treatment (Niu et al., 2010). In *A. commutatum*, both structural and regulatory genes were expressed at a high level under light conditions compared with the significant reduction in dark conditions. It can be seen that light is a vital environmental factor for the red coloration of *A. commutatum* leaf. Further studies are needed to reveal the potential mechanism of how light regulates anthocyanin biosynthesis in *A. commutatum*.

## Conclusions

In this study, novel R2R3-MYB and bHLH transcriptional factors were identified in *A. commutatum* “Red Valentine” leaves and named *AcMYB1* and *AcbHLH1*. Expression pattern analysis showed that the transcript abundances of *AcMYB1* and *AcbHLH1* are similar to those of several structural anthocyanin genes and correlate with anthocyanin distribution. AcbHLH1 interacts with AcMYB1 to form a transcriptional complex. Moreover, overexpression of *AcMYB1* in tobacco results in excessive accumulation of anthocyanins in tobacco leaves and other tissues and upregulates anthocyanin regulatory genes. Furthermore, light can significantly promote anthocyanin accumulation, and anthocyanin-related genes were strongly up-regulated in *A. commutatum* leaves. Therefore, we believe that *AcMYB1* is a key gene in regulating anthocyanin production in *A. commutatum* “Red Valentine.” Our study may be useful for modifying leaf color in ornamental breeding and provide a basis for further research and development in the plant breeding industry.

## Data Availability Statement

The datasets presented in this study can be found in online repositories. The names of the repository/repositories and accession number(s) can be found in the article/Supplementary Material.

## Author Contributions

JL, LF, and SZ conceived the research project, designed the research, and wrote the manuscript. JL and LF performed the research. JL, LF, LL, and GM analyzed the data. All authors read and approved the final manuscript.

## Funding

This work was funded by the Guangdong Key Technology Research and Development Program (2018B020202003) and the Guangdong Modern Agricultural Industry Technology System Program (2018LM2176 and 2022KJ121).

## Conflict of Interest

The authors declare that the research was conducted in the absence of any commercial or financial relationships that could be construed as a potential conflict of interest.

## Publisher's Note

All claims expressed in this article are solely those of the authors and do not necessarily represent those of their affiliated organizations, or those of the publisher, the editors and the reviewers. Any product that may be evaluated in this article, or claim that may be made by its manufacturer, is not guaranteed or endorsed by the publisher.

## References

[B1] Allan A. C. Hellens R. P. Laing W. A. (2008). MYB transcription factors that colour our fruit. Trends Plant Sci. 13, 99–102. 10.1016/j.tplants.2007.11.012 18280199

[B2] An C. H. Lee K. W. Lee S. H. Jeong Y. J. Woo S. G. Chun H.. (2015). Heterologous expression of IbMYB1a by different promoters exhibits different patterns of anthocyanin accumulation in tobacco. Plant Physiol. Bioch. 89, 1–10. 10.1016/j.plaphy.2015.02.002 25681576

[B3] Azuma A. Yakushiji H. Koshita Y. Kobayashi S. (2012). Flavonoid biosynthesis-related genes in grape skin are differentially regulated by temperature and light conditions. Planta 236, 1067–1080. 10.1007/s00425-012-1650-x 22569920

[B4] Bai Y. H. Pattanaik S. Patra B. Werkman J. R. Xie C. H. Yuan L. (2011). Flavonoid-related basic helix-loop-helix regulators, NtAn1a and NtAn1b, of tobacco have originated from two ancestors and are functionally active. Planta 234, 363–375. 10.1007/s00425-011-1407-y 21484270

[B5] Chen K. L. Du L. J. Liu H. L. Liu Y. L. (2019). A novel R2R3-MYB from grape hyacinth, MaMybA, which is different from MaAN2, confers intense and magenta anthocyanin pigmentation in tobacco. BMC Plant Biol. 19, 390. 10.1186/s12870-019-1999-0 31500571 PMC6734322

[B6] Chen K. L. Liu H. L. Lou Q. Liu Y. L. (2017). Ectopic expression of the grape hyacinth (*Muscari armeniacum*) R2R3-MYB transcription factor gene, MaAN2, induces anthocyanin accumulation in tobacco. Front. Plant Sci. 8, 965. 10.3389/fpls.2017.01722 28642775 PMC5462982

[B7] Cominelli E. Gusmaroli G. Allegra D. Galbiati M. Wade H. K. Jenkins G. I.. (2008). Expression analysis of anthocyanin regulatory genes in response to different light qualities in *Arabidopsis thaliana*. J. Plant Physiol. 165, 886–894. 10.1016/j.jplph.2007.06.010 17766004

[B8] D'Amelia V. Aversano R. Batelli G. Caruso I. Moreno M. C. Castro-Sanz A. B.. (2014). High AN1 variability and interaction with basic helix-loop-helix co-factors related to anthocyanin biosynthesis in potato leaves. Plant J. 80, 527–540. 10.1111/tpj.12653 25159050

[B9] Dooner H. K. Robbins T. P. Jorgensen R. A. (1991). Genetic and developmental control of anthocyanin biosynthesis. Annu. Rev. Genet. 25, 173–199. 10.1146/annurev.ge.25.120191.001133 1839877

[B10] Du B. G. Liang C. H. Chen Y. X. Lan T. W. (2013). Study on the factors influencing the bud induction of *Aglaonemum* cultured *in vitro*. Guangdong Agric. Sci. 40, 3. 10.16768/j.issn.1004-874x.2013.11.049

[B11] Espley R. V. Brendolise C. Chagne D. Kutty-Amma S. Green S. Volz R.. (2009). Multiple repeats of a promoter segment causes transcription factor autoregulation in red apples. Plant Cell 21, 168–183. 10.1105/tpc.108.059329 19151225 PMC2648084

[B12] Feller A. Machemer K Braun E. L. Grotewold E. (2011). Evolutionary and comparative analysis of MYB and bHLH plant transcription factors. Plant J. 66, 94–116. 10.1111/j.1365-313X.2010.04459.x 21443626

[B13] Feng C. Ding D. H. Feng C. Kang M. (2020). The identification of an R2R3-MYB transcription factor involved in regulating anthocyanin biosynthesis in *Primulina swinglei* flowers. Gene 752, 144788. 10.1016/j.gene.2020.144788 32439375

[B14] Gao J. M. Sun X. M. Zong Y. Yang S. P. Wang L. H. Liu B. L. (2020). Functional MYB transcription factor gene *HtMYB2* is associated with anthocyanin biosynthesis in *Helianthus tuberosus* L. BMC Plant Biol. 20, 247. 10.1186/s12870-020-02463-8 32487142 PMC7268318

[B15] Gao X. K. (2018). Preliminary study on tissue culture technique for *Aglaonema commutatun* ‘Big Apple'. J. Fujian Forest Sci. Technol. 45, 64–68. 10.13428/j.cnki.fjlk.2018.03.013

[B16] Gonzalez A. Zhao M. Leavitt J. M. Lloyd A. M. (2008). Regulation of the anthocyanin biosynthetic pathway by the TTG1/bHLH/Myb transcriptional complex in *Arabidopsis* seedlings. Plant J. 53, 814–827. 10.1111/j.1365-313X.2007.03373.x 18036197

[B17] Ha U. S. Bae W. J. Kim S. J. Yoon B. I. Hong S. H. Lee J. Y.. (2015). Anthocyanin induces apoptosis of DU-145 cells *in vitro* and inhibits xenograft growth of prostate cancer. Yonsei Med. J. 56, 16–23. 10.3349/ymj.2015.56.1.16 25510742 PMC4276751

[B18] Hichri I. Barrieu F. Bogs J. Kappel C. Delrot S. Lauvergeat V. (2011). Recent advances in the transcriptional regulation of the flavonoid biosynthetic pathway. J. Exp. Bot. 62, 2465–2483. 10.1093/jxb/erq442 21278228

[B19] Hou D. X. (2003). Potential mechanisms of cancer chemoprevention by anthocyanins. Curr. Mol. Med. 3, 149–159. 10.2174/1566524033361555 12630561

[B20] Hsu C. C. Chen Y. Y. Tsai W. C. Chen W. H. Chen H. H. (2015). Three R2R3-MYB transcription factors regulate distinct floral pigmentation patterning in *phalaenopsis* spp. Plant Physiol. 168, 175–U910. 10.1104/pp.114.254599 25739699 PMC4424010

[B21] Huang G. H. Liao X. Z. Han Q. Zhou Z. Z. Liang K. N. Li G. Y.. (2022). Integrated metabolome and transcriptome analyses reveal dissimilarities in the anthocyanin synthesis pathway between different developmental leaf color transitions in *Hopea hainanensis* (Dipterocarpaceae). Front. Plant Sci. 3, 830413. 10.3389/fpls.2022.830413 35310646 PMC8928120

[B22] Huang W. J. Khaldun A. B. M. Lv H. Y. Du L. W. Zhang C. J. Wang Y. (2016). Isolation and functional characterization of a R2R3-MYB regulator of the anthocyanin biosynthetic pathway from *Epimedium sagittatum*. Plant Cell Rep. 35, 883–894. 10.1007/s00299-015-1929-z 26849670

[B23] Huang W. J. Lv H. Y. Wang Y. (2017). Functional characterization of a novel R2R3-MYB transcription factor modulating the flavonoid biosynthetic pathway from *Epimedium sagittatum*. Front. Plant Sci. 8, 1274. 10.3389/fpls.2017.01274 28769969 PMC5515856

[B24] Hughes N. M. Neufeld H. S. Burkey K. O. (2005). Functional role of anthocyanins in high-light winter leaves of the evergreen herb *Galax urceolata*. New Phytol. 168, 575–587. 10.1111/j.1469-8137.2005.01546.x 16313641

[B25] Koes R. Verweij W. Quattrocchio F. (2005). Flavonoids: a colorful model for the regulation and evolution of biochemical pathways. Trends Plant Sci. 10, 236–242. 10.1016/j.tplants.2005.03.002 15882656

[B26] Lepiniec L. Debeaujon I. Routaboul J. M. Baudry A. Pourcel L. Nesi N.. (2006). Genetics and biochemistry of seed flavonoids. Annu. Rev. Plant Biol. 57, 405–430. 10.1146/annurev.arplant.57.032905.105252 16669768

[B27] Li C. H. Qiu J. Yang G. S. Huang S. R. Yin J. M. (2016). Isolation and characterization of a R2R3-MYB transcription factor gene related to anthocyanin biosynthesis in the spathes of *Anthurium andraeanum* (Hort.). Plant Cell Rep. 35, 2151–2165. 10.1007/s00299-016-2025-8 27424029

[B28] Li X. Li Y. Zhao M. H. Hu Y. B. Meng F. J. Song X. H.. (2021). Molecular and metabolic insights into anthocyanin biosynthesis for leaf color change in chokecherry (*Padus virginiana*). Int. J. Mol. Sci. 22, 10697. 10.3390/ijms221910697 34639038 PMC8509056

[B29] Li Y. Q. Shan X. T. Tong L. N. Wei C. Lu K. Y. Li S. Y.. (2020). The conserved and particular roles of the R2R3-MYB regulator FhPAP1 from *Freesia hybrida* in flower anthocyanin biosynthesis. Plant Cell Physiol. 61, 1365–1380. 10.1093/pcp/pcaa065 32392327

[B30] Lightbourn G. J. Griesbach R. J. Novotny J. A. Clevidence B. A. Rao D. D. Stommel J. R. (2008). Effects of anthocyanin and carotenoid combinations on foliage and immature fruit color of *Capsicum annuum* L. J. Hered. 99, 105–111. 10.1093/jhered/esm108 18222931

[B31] Livak K. J. Schmittgen T. D. (2001). Analysis of relative gene expression data using real-time quantitative PCR and the 2(-Delta Delta C(T)) method. Methods 25, 402–408. 10.1006/meth.2001.1262 11846609

[B32] Martin C. Butelli E. Petroni K. Tonelli C. (2011). How can research on plants contribute to promoting human health? Plant Cell 23, 1685–1699. 10.1105/tpc.111.083279 21586682 PMC3123949

[B33] Mol J. Grotewold E. Koes R. (1998). How genes paint flowers and seeds. Trends Plant Sci. 3, 212–217. 10.1016/S1360-1385(98)01242-4

[B34] Nakatsuka A. Yamagishi M. Nakano M. Tasaki K. Kobayashi N. (2009). Light-induced expression of basic helix-loop-helix genes involved in anthocyanin biosynthesis in flowers and leaves of Asiatic hybrid lily. Sci. Hortic. 121, 84–91. 10.1016/j.scienta.2009.01.008

[B35] Nesi N. Debeaujon I. Jond C. Pelletier G. Caboche M. Lepiniec L. (2000). The *TT8* gene encodes a basic helix-loop-helix domain protein required for expression of *DFR* and *BAN* genes in Arabidopsis siliques. Plant Cell 12, 1863–1878. 10.1105/tpc.12.10.1863 11041882 PMC149125

[B36] Niu S. S. Xu C. J. Zhang W. S. Zhang B. Li X. Lin-Wang K.. (2010). Coordinated regulation of anthocyanin biosynthesis in Chinese bayberry (*Myrica rubra*) fruit by a R2R3 MYB transcription factor. Planta 231, 887–899. 10.1007/s00425-009-1095-z 20183921

[B37] Nuraini L. Ando Y. Kawai K. Tatsuzawa F. Tanaka K. Ochiai M.. (2020). Anthocyanin regulatory and structural genes associated with violet flower color of *Matthiola incana*. Planta 251, 61. 10.1007/s00425-020-03351-z 32036464

[B38] Palapol Y. Ketsa S. Kui L. W. Ferguson I. B. Allan A. C. (2009). A MYB transcription factor regulates anthocyanin biosynthesis in mangosteen (*Garcinia mangostana* L.)fruit during ripening. Planta 229, 1323–1334. 10.1007/s00425-009-0917-3 19306102

[B39] Patra B. Schluttenhofer C. Wu Y. M. Pattanaik S. Yuan L. (2013). Transcriptional regulation of secondary metabolite biosynthesis in plants. Biochim. Biophys. Acta 1829, 1236–1247. 10.1016/j.bbagrm.2013.09.006 24113224

[B40] Pattanaik S. Kong Q. Zaitlin D. Werkman J. R. Xie C. H. Patra B.. (2010). Isolation and functional characterization of a floral tissue-specific R2R3 MYB regulator from tobacco. Planta 231, 1061–1076. 10.1007/s00425-010-1108-y 20157728

[B41] Pattanaik S. Xie C. H. Yuan L. (2008). The interaction domains of the plant Myc-like bHLH transcription factors can regulate the transactivation strength. Planta 227, 707–715. 10.1007/s00425-007-0676-y 18075757

[B42] Payne C. T. Zhang F. Lloyd A. M. (2000). *GL3* encodes a bHLH protein that regulates trichome development in arabidopsis through interaction with GL1 and TTG1. Genetics 156, 1349–1362. 10.1093/genetics/156.3.1349 11063707 PMC1461316

[B43] Petroni K. Tonelli C. (2011). Recent advances on the regulation of anthocyanin synthesis in reproductive organs. Plant Sci. 181, 219–229. 10.1016/j.plantsci.2011.05.009 21763532

[B44] Qin J. Zhao C. Z. Wang S. W. Gao N. Wang X. X. Na X. F.. (2022). PIF4-PAP1 interaction affects MYB-bHLH-WD40 complex formation and anthocyanin accumulation in Arabidopsis. J. Plant Physiol. 268, 153558. 10.1016/j.jplph.2021.153558 34798465

[B45] Schaefer H. M. Schaefer V. Levey D. J. (2004). How plant-animal interactions signal new insights in communication. Trends Ecol. Evol. 19, 577–584. 10.1016/j.tree.2004.08.00318031220

[B46] Shimizu Y. Maeda K. Kato M. Shimomura K. (2011). Co-expression of *GbMYB1* and *GbMYC1* induces anthocyanin accumulation in roots of cultured *Gynura bicolor* DC. plantlet on methyl jasmonate treatment. Plant Physiol. Bioch. 49, 159–167. 10.1016/j.plaphy.2010.11.006 21123079

[B47] Spelt C. Quattrocchio F. Mol J. N. M. Koes R. (2000). anthocyanin1 of petunia encodes a basic helix-loop-helix protein that directly activates transcription of structural anthocyanin genes. Plant Cell 12, 1619–1631. 10.1105/tpc.12.9.1619 11006336 PMC149074

[B48] Tian J. Peng Z. Zhang J. Song T. T. Wan H. H. Zhang M. L.. (2015). McMYB10 regulates coloration via activating *McF3'H* and later structural genes in ever-red leaf crabapple. Plant Biotechnol. J. 13, 948–961. 10.1111/pbi.12331 25641214

[B49] Vimolmangkang S. Han Y. P. Wei G. C. Korban S. S. (2013). An apple MYB transcription factor, *MdMYB3*, is involved in regulation of anthocyanin biosynthesis and flower development. BMC Plant Biol. 13, 176. 10.1186/1471-2229-13-176 24199943 PMC3833268

[B50] Waadt R. Schmidt L. K. Lohse M. Hashimoto K. Bock R. Kudla J. (2008). Multicolor bimolecular fluorescence complementation reveals simultaneous formation of alternative CBL/CIPK complexes *in planta*. Plant J. 56, 505–516. 10.1111/j.1365-313X.2008.03612.x 18643980

[B51] Walter M. Chaban C. Schutze K. Batistic O. Weckermann K. Nake C.. (2004). Visualization of protein interactions in living plant cells using bimolecular fluorescence complementation. Plant J. 40, 428–438. 10.1111/j.1365-313X.2004.02219.x 15469500

[B52] Wang H. H. Wang X. Q. Song W. M. Bao Y. Jin Y. L. Jiang C. M.. (2019). PdMYB118, isolated from a red leaf mutant of *Populus deltoids*, is a new transcription factor regulating anthocyanin biosynthesis in poplar. Plant Cell Rep. 38, 927–936. 10.1007/s00299-019-02413-1 31147728

[B53] Winkel-Shirley B. (2001). Flavonoid biosynthesis. A colorful model for genetics, biochemistry, cell biology, and biotechnology. Plant Physiol. 126, 485–493. 10.1104/pp.126.2.485 11402179 PMC1540115

[B54] Wrolstad R. E. Culbertson J. D. Cornwell C. J. Mattick L. R. (1982). Detection of adulteration in blackberry juice concentrates and wines. J. Assoc. Off. Anal. Chem. 65, 1417–1423. 10.1093/jaoac/65.6.1417 7174584

[B55] Xu H. F. Zou Q. Yang G. X. Jiang S. H. Fang H. C. Wang Y. C.. (2020). MdMYB6 regulates anthocyanin formation in apple both through direct inhibition of the biosynthesis pathway and through substrate removal. Hortic Res. 7, 72. 10.1038/s41438-020-0294-4 32377362 PMC7195469

[B56] Xu W. J. Dubos C. Lepiniec L. (2015). Transcriptional control of flavonoid biosynthesis by MYB-bHLH-WDR complexes. Trends Plant Sci. 20, 176–185. 10.1016/j.tplants.2014.12.001 25577424

[B57] Xu W. J. Grain D. Bobet S. Le Gourrierec J. Thevenin J. Kelemen Z.. (2014). Complexity and robustness of the flavonoid transcriptional regulatory network revealed by comprehensive analyses of MYB-bHLH-WDR complexes and their targets in Arabidopsis seed. New Phytol. 202, 132–144. 10.1111/nph.12620 24299194

[B58] Yin X. J. Zhang Y. B. Zhang L. Wang B. H. Zhao Y. D. Irfan M.. (2021). Regulation of MYB transcription factors of anthocyanin synthesis in lily flowers. Front. Plant Sci. 12, 761668. 10.3389/fpls.2021.761668 34925411 PMC8672200

[B59] Yoo S. D. Cho Y. H. Sheen J. (2007). Arabidopsis mesophyll protoplasts: a versatile cell system for transient gene expression analysis. Nat. Protoc. 2, 1565–1572. 10.1038/nprot.2007.199 17585298

[B60] Zhang B. Hu Z. L. Zhang Y. J. Li Y. L. Zhou S. Chen G. P. (2012). A putative functional MYB transcription factor induced by low temperature regulates anthocyanin biosynthesis in purple kale (*Brassica Oleracea* var. acephala f. tricolor). Plant Cell Rep. 31, 281–289. 10.1007/s00299-011-1162-3 21987119

[B61] Zhang M. M. Ji L. S. Xue H. Yang Y. T. Wu C. A. Zheng C. C. (2007). High transformation frequency of tobacco and rice via *Agrobacterium*-mediated gene transfer by flanking a tobacco matrix attachment region. Physiol. Plant. 129, 644–651. 10.1111/j.1399-3054.2006.00779.x

[B62] Zhang Y. Z. Xu S. Z. Cheng Y. W. Peng Z. F. Han J. M. (2018). Transcriptome profiling of anthocyanin-related genes reveals effects of light intensity on anthocyanin biosynthesis in red leaf lettuce. PeerJ 6, e4607. 10.7717/peerj.4607 29666761 PMC5900932

[B63] Zhao J. Dixon R. A. (2010). The 'ins' and 'outs' of flavonoid transport. Trends Plant Sci. 15, 72–80. 10.1016/j.tplants.2009.11.006 20006535

[B64] Zhao J. T. Chen L. H. Ma A. Wang D. Lu H. Chen L.. (2022). R3-MYB transcription factor LcMYBx from Litchi chinensis negatively regulates anthocyanin biosynthesis by ectopic expression in tobacco. Gene 812, 146105. 10.1016/j.gene.2021.146105 34896231

[B65] Zhou Y. Zhou H. Kui L. W. Vimolmangkang S. Espley R. V. Wang L.. (2014). Transcriptome analysis and transient transformation suggest an ancient duplicated MYB transcription factor as a candidate gene for leaf red coloration in peach. BMC Plant Biol. 14, 388. 10.1186/s12870-014-0388-y 25551393 PMC4302523

[B66] Zimmermann I. M. Heim M. A. Weisshaar B. Uhrig J. F. (2004). Comprehensive identification of *Arabidopsis thaliana* MYB transcription factors interacting with R/B-like BHLH proteins. Plant J. 40, 22–34. 10.1111/j.1365-313X.2004.02183.x 15361138

