# Supplementary material for: AcMYB1 Interacts With AcbHLH1 to Regulate Anthocyanin Biosynthesis in Aglaonema commutatum
Source: Front Plant Sci. 2022 Jul 19;13:886313. doi: 10.3389/fpls.2022.886313 (PMC9344012; doi:10.3389/fpls.2022.886313)
Supplement: Supplementary file 1 [file Data_Sheet_1.docx]

**AcMYB1 interacts with AcbHLH1 to regulate anthocyanin biosynthesis in *Aglaonema commutatum***

Ji Li^a,b^, Kunlin Wu^a^, Lin Li^a^, Guohua Ma^a^, Lin Fang^a,c*^and Songjun Zeng^a,d*^

**Supplementary Data**

**Supplementary Figures**

**Figure S1:** **Sequence alignment analyses of AcbHLH1.** The MYB-interating region are shown with red line and the conserved bHLH domain is indicated in the green line.

**
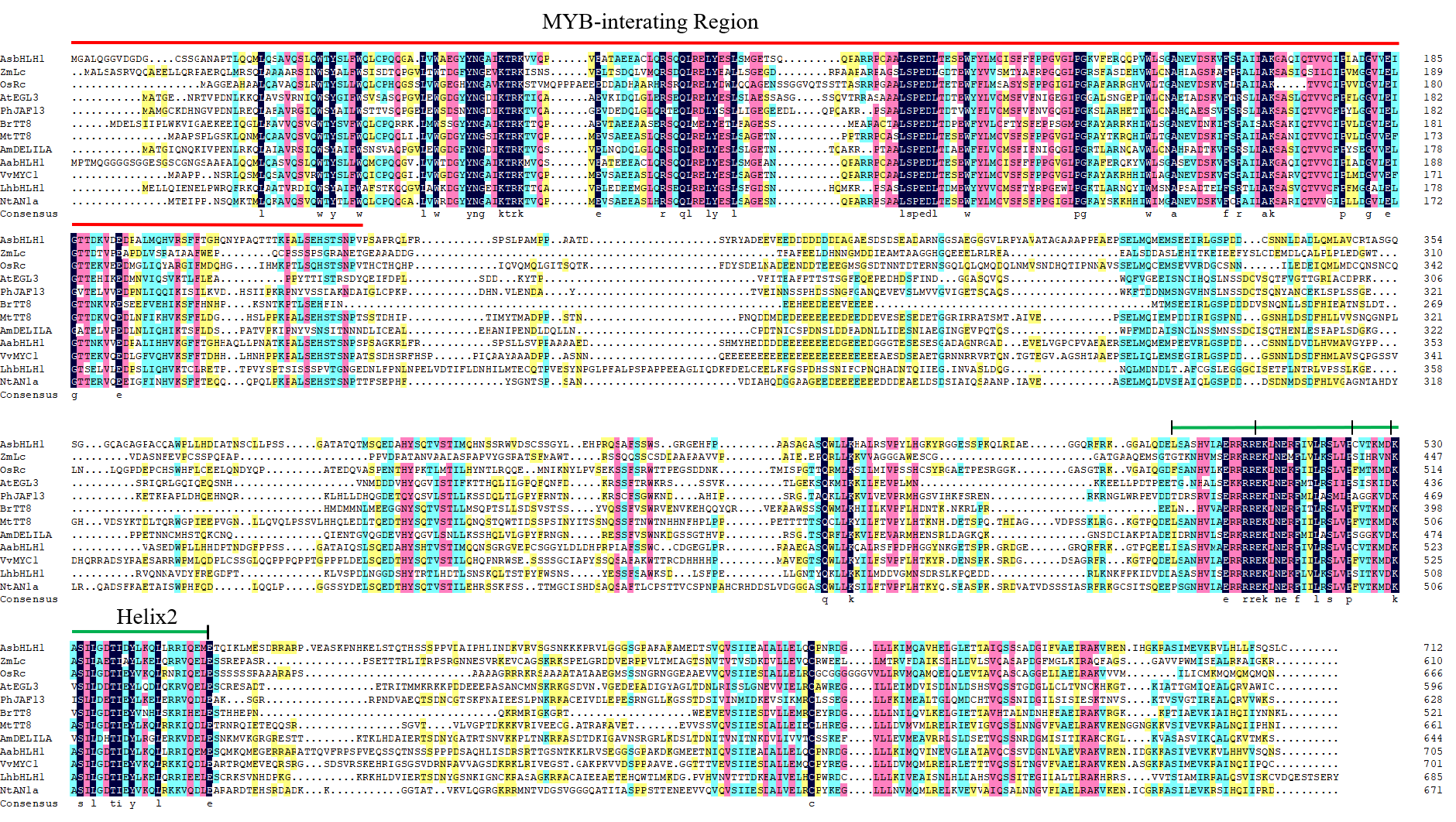
**

**Figure S2:** **PCR validation of transgenic *N. tabacum* plants over expression *AcMYB1* lines.** M: 2000 bp DNA marker; Lane 1: positive control, Lane 2: negative control, Lane 3-20: transgenic line 1 to 18 of over-expression AcMYB1.

**
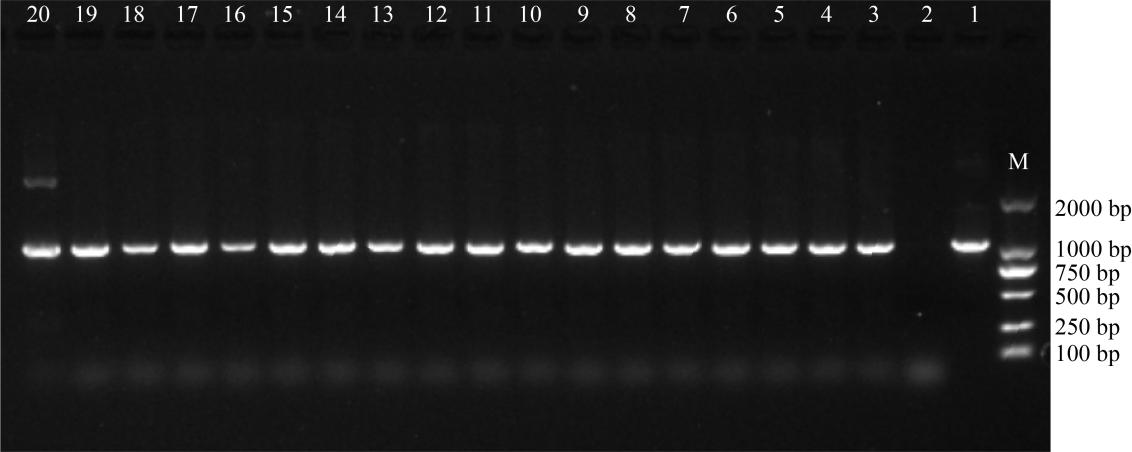
**

**Figure S3：Vegetative and reproductive tissues of the OE-*AcMYB1* lines.** A and B: Vegetative tissues; C and D: Reproductive tissues; E: Transgenic tobaccos in the greenhouse.

**
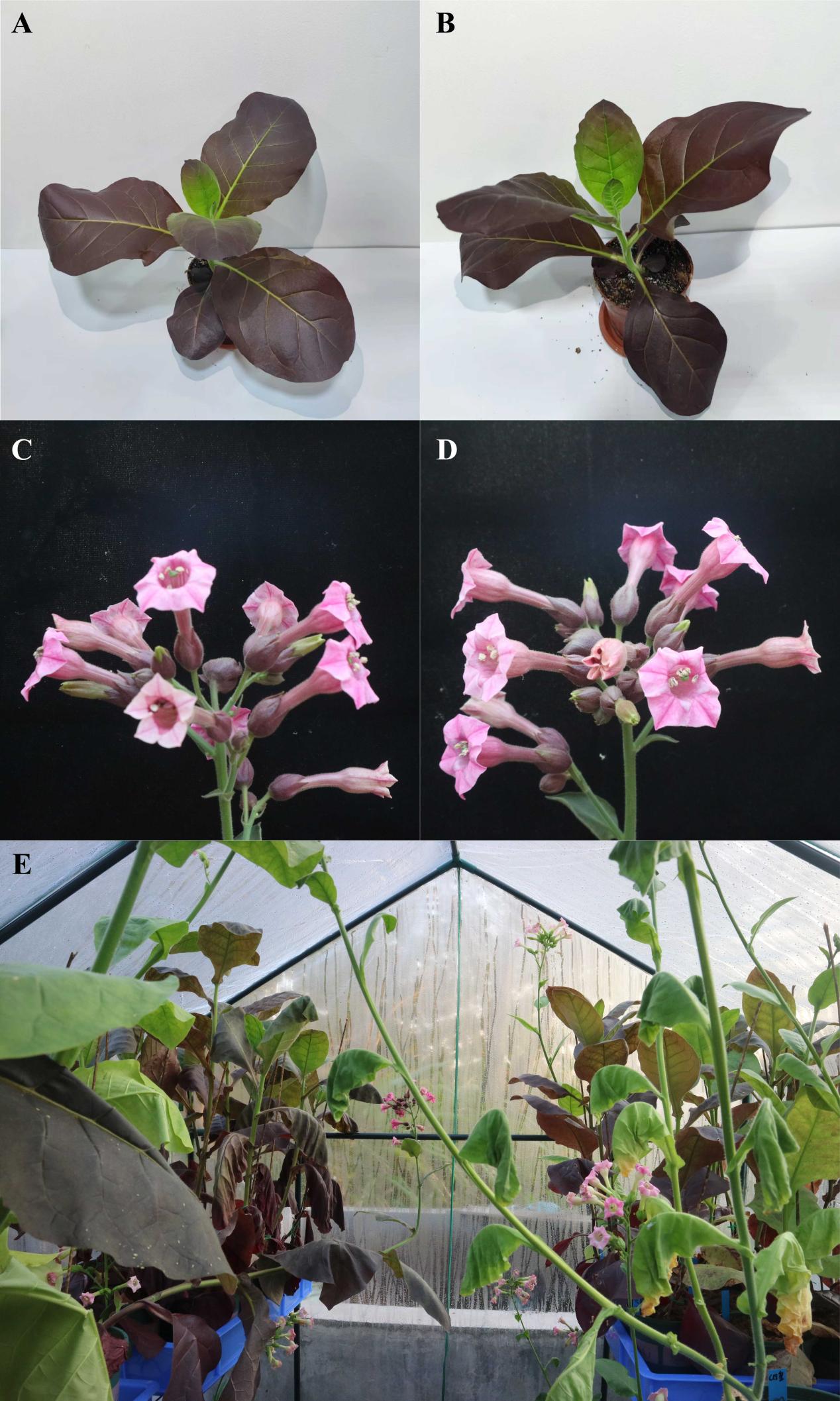
**

**Supplementary Tables**

**Table S1:** Primers used for qRT-PCR

| Primer name | Primer sequence |
| --- | --- |
| *AcCHS1* | Fw: 5’- CCGTACTCCTTGAGCACCTC -3’ |
|  | Rv: 5’- GGGCATCAACGACTGGAACT -3’ |
| *AcCHS2* | Fw: 5’- CCACCAGGCTCTTCTCGATG -3’ |
|  | Rv: 5’- CCCTCTTTGAGCTGGTCTCG -3’ |
| *AcCHI* | Fw: 5’- GTGTCGGACGTTCAGGTTGA -3’ |
|  | Rv: 5’- CCCGATGGCGGTGAACTTTA -3’ |
| *AcF3H* | Fw: 5’- GAGAAGCTCCGCTTCGACAT -3’ |
|  | Rv: 5’- GCGTAGCGGGTACGAGAAAT -3’ |
| *AcF3'H* | Fw: 5’- ATGACCTCGAAGTGGTTCCC -3’ |
|  | Rv: 5’- ACTCTCCTCGTGAACGTGTG -3’ |
| *AcDFR1* | Fw: 5’- GCTGAGCGTATTGAGCTCCT -3’ |
|  | Rv: 5’- CATCCAGCCGGTCATCTTGA -3’ |
| *AcDFR3* | Fw: 5’- AGATGTACCTTCCGCTGGCT -3’ |
|  | Rv: 5’- TCTAATTTGGATGGCCCCCTG -3’ |
| *AcANS* | Fw: 5’- GAAGAGACGAGGGAGTTCGG -3’ |
|  | Rv: 5’- GCACCGCGGGTAGTAGTTTA -3’ |
| *AcUFGT1* | Fw: 5’- CTCACCAAGGCTGTCGACCT -3’ |
|  | Rv: 5’- CGAAGACGGAGAAGAAGACA -3’ |
| *AcUFGT2* | Fw: 5’- CCTTCCGAGGTCGAGGAGAT -3’ |
|  | Rv: 5’- GCTCTAGCTCGGAGAACGTG -3’ |
| *AcMYB1* | Fw: 5’- GGCTCAACTACCTCAGACCG -3’ |
|  | Rv: 5’- AAGGACCACCTGTTGCCAAT -3’ |
| *AcbHLH1* | Fw: 5’- TGGCTTTCAGGGGCAAATGA -3’ |
|  | Rv: 5’- GTCCTCGTCCACCTTATCGG -3’ |
| *AcEF-1α* | Fw: 5’- ATGAAGCCGAGCGTGGTATC -3’ |
|  | Rv: 5’- TACGAAGAGCAGCCGTAACC -3’ |
| *NtPAL* | Fw: 5’- ATTGAGGTCATCCGTTCTGC -3’ |
|  | Rv: 5’- ACCGTGTAACGCCTTGTTTC -3’ |
| *NtCHS* | Fw: 5’- TGACACCCACTTGGATAGTTTAG -3’ |
|  | Rv: 5’- CGACCTCTGGAATTGGATCAG -3’ |
| *NtCHI* | Fw: 5’- CTTTTCTCGCCGCTAAATG -3’ |
|  | Rv: 5’- TTTCTGCCACCTTCTCTG -3’ |
| *NtF3H* | Fw: 5’- CAAGGCATGTGTGGATATGG -3’ |
|  | Rv: 5’- TGTGTCGTTTCAGTCCAAGG -3’ |
| *NtF3'H* | Fw: 5’- AGGCTCAACACTTCTCGT -3’ |
|  | Rv: 5’- CATCAACTTTGGGCTTCT -3’ |
| *NtF3'5'H* | Fw: 5’- TGCACACTATGGACCACGTT -3’ |
|  | Rv: 5’- CCTTCTCGGCTCATTTCGGA -3’ |
| *NtDFR* | Fw: 5’- AACCAACAGTCAGGGGAATG -3’ |
|  | Rv: 5’- TTGGACATCGACAGTTCCAG -3’ |
| *NtANS* | Fw: 5’- TGGCGTTGAAGCTCATACTG -3’ |
|  | Rv: 5’- GGAATTAGGCACACACTTTGC -3’ |
| *NtUFGT* | Fw: 5’- GAGTGCATTGGATGCCTTTT -3’ |
|  | Rv: 5’- CCAGCTCCATTAGGTCCTTG -3’ |
| *NtAn1a* | Fw: 5’- ACCATTCTCGAACACCGAAG -3’ |
|  | Rv: 5’- TGCTAGGGCACAATGTGAAG -3’ |
| *NtAn1b* | Fw: 5’- CTTGAACACTTCTCAAACCGA -3’ |
|  | Rv: 5’- TGCTAGGGCACAATGTGAAG -3’ |
| *NtActin* | Fw: 5’- CCTGAGGTCCTTTTCCAACCA -3’ |
|  | Rv: 5’- GGATTCCGGCAGCTTCCATT -3’ |
| *NbCHS* | Fw: 5’- ATAGTTTGGTTGGGCAAGCC -3’ |
|  | Rw: 5’- AGACAAGCTGGAACAAAGGC -3’ |
| *NbCHI* | Fw: 5’- CCCAATGGTGCAACTGTAAAGG -3’ |
|  | Rw: 5’- TGCCAAGCATGCTCTTCTTC -3’ |
| *NbF3H* | Fw: 5’- TGCATGAAAACCGGTTCGTC -3’ |
|  | Rw: 5’- TCGGCCCTACGTGATTTGATAC -3’ |
| *NbDFR* | Fw: 5’- ATTCATCTGCGCATCCCATC -3’ |
|  | Rw: 5’- ACCACAGGCAAGTCCTTATCG -3’ |
| *NbANS* | Fw: 5’- TCCTCCACAATATGGTGCCTG -3’ |
|  | Rw: 5’- GGGTGTCCCCAATATGCATG -3’ |
| *NbUFGT* | Fw: 5’- CCAACACCTCAATCTTCTCTAAATC -3’ |
|  | Rw: 5’- TCCTCTGCCTCTTTCATAACTTTCT -3’ |
| *NbActin* | Fw: 5’- TGCAAAGACCAGCTCTTCTG -3’ |
|  | Rw: 5’- ATTCCTGCAGCTTCCATTCC -3’ |

**Table S2:** Genbank accession numbers referred in this study.

| Type | Protein name | Accession numbers | Species |
| --- | --- | --- | --- |
| MYB | PhAN2 | AAF66727 | *Petunia hybrida* |
|  | AtPAP1 | AAG42001 | *Arabidopsis thaliana* |
|  | AtPAP2 | AAG42002 | *Arabidopsis thaliana* |
|  | IbMYB1 | BAF45114 | *Ipomoea batatas* |
|  | ZmC1 | AAA33482 | *Zea mays* |
|  | StAN1 | AAX53087 | *Solanum tuberosum* |
|  | StAN2 | AAX53091 | *Solanum tuberosum* |
|  | NtAN2 | ACO52472 | *Nicotiana tabacum* |
|  | AaMYB2 | AML84515 | *Anthurium andraeanum* |
|  | MaAN2 | ASF20090 | *Muscari armeniacum* |
|  | LhMYB6 | BAJ05399 | *Lilium hybrid division Ⅰ* |
|  | LhMYB12 | BAJ05398 | *Lilium hybrid division Ⅰ* |
|  | OsMYB | CAA75509 | *Oryza sativa* |
|  | ZmPl | AAB67720 | *Zea mays* |
|  | DhMYB2 | AQS79852 | *Dendrobium hybrid cultivar* |
|  | PeMYB11 | QCS14308 | *Phalaenopsis equestris* |
|  | OgMYB1 | ABS58501 | *Oncidium gower* |
|  | ROSEA1 | AKB94073 | *Antirrhinum majus* |
|  | SlMYB | AAQ55181 | *Solarium lycopersicum* |
|  | AcMYB1 | OM688334 | *Aglaonema commutatum* ‘Red Valentine’ |
| bHLH | ZmLc | AAA33504 | *Zea mays* |
|  | OsRc | ABB17166 | *Oryza sativa* |
|  | AtEGL3 | NP_176552 | *Arabidopsis thaliana* |
|  | AtGL3 | NP_680372 | *Arabidopsis thaliana* |
|  | AtTT8 | CAC14865 | *Arabidopsis thaliana* |
|  | GhMYC1 | CAA07615 | *Gerbera hybrida* |
|  | PhAN1 | AAG25927.1 | Petunia hybrida |
|  | PhJAF13 | AAC39455 | Petunia hybrida |
|  | BrTT8 | AEA03281.1 | *Brassica rapa* |
|  | TrAN1 | AIT76559 | *Trifolium repens* |
|  | MtEGL3 | KEH21065 | *Medicago truncatula* |
|  | MtTT8 | AKN79606 | *Medicago truncatula* |
|  | GbMYC1 | BAJ17663 | *Gynura bicolor* |
|  | AmDELILA | AAA32663 | *Antirrhinum majus* |
|  | InbHLH1 | BAE94393 | *Ipomoea nil* |
|  | ZmIN1 | AAB03841 | *Zea mays* |
|  | AabHLH1 | AZS49191 | *Anthurium andraeanum* |
|  | VvMYC1 | ACC68685 | Vitis vinifera |
|  | LhbHLH1 | BAE20057 | *Lilium hybrid division Ⅰ* |
|  | MrbHLH1 | AGO58372 | *Myrica rubra* |
|  | NtAn1a | AEE99257 | *Nicotiana tabacum* |
|  | NtAn1b | AEE99258 | *Nicotiana tabacum* |
|  | AcbHLH1 | OM688322 | Aglaonema commutatum ‘Red Valentine’ |

**Table S3:** Primers used for cloning and plasmid construction.

| **Primers used for gene cloning** | |
| --- | --- |
| *AcMYB1*-F | 5’- ATGCTCCGCATCCTCCGAAT-3’ |
| *AcMYB1*-R | 5’- TTATAACAGCCGCATAAGGC-3’ |
| *AcbHLH1*-F | 5’- ATGGGCGCCCTGCAGGGTGGCG-3’ |
| *AcbHLH1*-R | 5’- TTAACACAAGCTTTGAGAAAAT-3’ |
| **Primers used for subcellular localization (Infusion)** | |
| pSAT6-EYFP-N1-*AcMYB1*-F | 5’- CTCAAGCTTCGAATTCATGCTCCGCATCCTCCGAATATGC-3’ |
| pSAT6-EYFP-N1-*AcMYB1*-R | 5’- CTCACCATCAGGATCGTTAACAGCCGCATAAGGCTAGGG-3’ |
| pSAT6-EYFP-N1-*AcbHLH1*-F | 5’- CTCAAGCTTCGAATTCATGGGCGCCCTGCAGGGTG-3’ |
| pSAT6-EYFP-N1-*AcbHLH*-R | 5’- CTCACCATCAGGATCGTACACAAGCTTTGAGAAAAT-3’ |
| **Primers used for Bimolecular Fluorescence Complementation (Infusion)** | |
| pSPYNE/*AcMYB1*-F | 5’- CACGGGGGACTCTAGATGCTCCGCATCCTCCGAATATGC-3’ |
| pSPYNE/*AcMYB1*-R | 5’- TACTATCGATGGATCCTAACAGCCGCATAAGGCTAGGG-3’ |
| pSPYCE/*AcbHLH1*-F | 5’- CACGGGGGACTCTAGATGGGCGCCCTGCAGGGTGGCG-3’ |
| pSPYCE/*AcbHLH1*-R | 5’- TACTATCGATGGATCCACACAAGCTTTGAGAAAAT-3’ |
| **Primers used for Y2H (Infusion)** | |
| pGADT7-*AcMYB1*-F | 5’- GGAGGCCAGTGAATTCATGCTCCGCATCCTCCGAATATGC-3’ |
| pGADT7-*AcMYB1*-R | 5’- CGAGCTCGATGGATCTTATAACAGCCGCATAAGGCTAGGG-3’ |
| pGBKT7-*AcbHLH1*-F | 5’- CATGGAGGCCGAATTCATGGGCGCCCTGCAGGGT-3’ |
| pGBKT7-*AcbHLH1*-R | 5’- GCAGGTCGACGGATCTTAACACAAGCTTTGAGAAAATAGA-3’ |
| **Primers used for overexpression in tobacco (Infusion)** | |
| pGreen-C17-*AcMYB1*-F | 5’- GCTTGATATCGAATTCATGCTCCGCATCCTCCGAATATGC-3’ |
| pGreen-C17-*AcMYB1*-R | 5’- CAGCGAATTATCTAGATAACAGCCGCATAAGGCTAGGG-3’ |

**Table S4:** The complete protein sequences used in this work.

| Gene name | Protein sequence |
| --- | --- |
| *AcEF-1α* | *>AcEF-1α*  ATGCCTCGTAAAGTTAGCTATGGGCTTGATTGTGATGATGAGTATGGTGATTATGATGACTATGATGACTATGATTACGACTATGATGTAGATGATGCGGGGAAAACATCTCACATAGCTCTGAGTCATCAAGTTAAGAAGTCTGGACTTTGGCGATGCTCCATTTGCACTTACGACAATGATGAAAGTTTATCCTTTTGTGATATTTGTGGAGTTATTCGGGAATCCTTTGCCAACCTTGCCATTAGTGGTAAAGAAAAAGTTGATGCTGTTGACAAAATTTATGGAGCTTCGATAATGGCCAAGTCTCTTTTTTCATCAATTCCTTCAGGGGGCCCCAAAGCTGCTCCTGCCTTTCAGCAACTAAATGAGGAATCTTTGAACATCAAGGAAAATGACTTCCGCGGCAACATAAATCTTCAAGGATGTTTTGATGATCCACAGGCCTTTTTGGCTCCTGACAGCAATGCCCATAAGAACATAGCTTCATTCAAGTTTGATAGTCCATCTCCGGATGATATTGTTTCTTCAGGGAAGAATGCATCGAGAGTTGCCCAAAAAGCTAGGGCTACATCTGTCATCACCAGGAGCAAAGATGTTGCTGAAGATTTGCCCATTGCCAAAGTGTTGGAGTGTTCATCTGGAATAACATTTAAAGATCGTCAACATAATGTCAACGAAAGCAGTAGTGCAGTGGACAGTGATGTCAGATCAAAGATTCCAGCGAGCAACTTACAGGATTTGGATTCATATAAAAACTCTGGAAGTCAAAAGAAAATTATGAACAGAGATATGAGTGTATCTGCTCAGTACAAGCCAGAGAAATGGATGTTACCTGATCAGGAGCAGGGGGCCTTGGTTCAATTAAATCTTGCAATTGTTGGCCATGTGGATTCTGGAAAATCAACATTGTCAGGGAGATTGCTACATCAGTTGGGGCGCATATCTCAGAAAGAAATGCACAAATATGAGAAAGAGGCTAAAGAGAAGGGAAAAGGATCATTCGCTTATGCTTGGGCAATGGATGAAAGTGCTGAAGAGAGGGAGAGGGGTATCACTATGACAGTGGCAGTTGCTTATTTTGATACCCAGAAATATCATATTGTGTTGCTTGATTCCCCTGGCCATAAGGACTTTGTTCCAAATCTGATCTCGGGTGCTACACAAGCAGATGCTGCTATTCTTGTTATAGATTCATCTATTGGTTCCTTTGAAGCAGGTATGGAGGGTGGAGGGCAGACACGGGAACATGCACAACTTGTTAGAAGTTTTGGTGTGGAGCAGGTAATTGTTGCTGTTAACAAAATGGACACTGTGGAGTACTCGAAAGAACGATTTGATTCTATAAAGGTGCAACTTGGTTTGTTCCTCCGTACCTGTGGATTCAAAGAATCATCTATCCTCTGGATCCCTCTTAGTGCCATTGAGAATCAAAATCTGGTAAAAGCAGCCGCTGATGCCCGTCTGTCTTGTTGGTATGATGGCCCATCTCTGTTGGATGCGATCAATTCTCTTCAACCTCCCTTGAGGGATGTCTCTAAGCCTCTACGTTTGCCTATATGTGATGTTATTAAGTCACGTTCTGTGGGACATGTGTCAGCTAGTGGCAAATTGGAGACAGGAGCGATTCGAAATGGTACTAAGGTTCTAGTTATGCCTTCTGGGGATACAGCCACGGTGCGATCTATTGAGCGTGATTCTTGTATATGCAGTGTAGCAAGAGCTGGTGACAATGTGGCTGTTGTTTTGCAAGGCATTGACACAGGCAATGTGATGTCAGGGAGTGTTTTGTGTCACCCAGAGTTCCCTGTCCCTGTTGCAACTTGTTTGGAGCTGAAAATCCTTCTCCTGGATGTAGCTACACCAATTTTAGTAGGGTCAGAGGTCGAATTTCACATACACCATGTCCGGGAGGCTGCAAGAGTGGCAAAGATTTCATCAGTAATAGACCCCAAAACGGGCAAGGTCTCCAAGAAGGCGGCTCGTTTTCTTTCAGCAAAACAAAGTGCCGTGATTGAGGTTGACCTTGATGGGGCTGTTTGTGTGGAGGAATTTTCCAAGTGCCGGGCTCTTGGGAGGGTGTTTTTACGGGCATCCGGGAACACAGTTGCTGTCGGTATTGTAACCAGGGTTATCCAGCAGGAGTAA |
| *AcMYB1* | >*AcMYB1*  ATGCTCCGCATCCTCCGAATATGCATCAGAGATGGCCAAGTTATCCCTCCCGTCTATAAATCCAAGGACACCGCAGAAGTAGCTGCGAGGAGCGTAGAACTTGCTACGATGGCTGGCGCACATGTGCATCACTTCACGGGGAGTGGAGATATCCGAAAGGGTGCATGGGCGCCTGAGGAAGACAACCTTCTCAGAGACTGCATCAAAAAGTATGGTGAAGGAAAGTGGCACCTTGTTCCAGATAGGGCAGGTCTGAGGCGCTGCCGCAAAAGCTGCCGCTTGCGGTGGCTGAACTATCTCAAACCCAACATTAAGCGAGGGAAATTTCAAGAAGATGAAGTAGATCTCATATTGAGGCTCCATCACCTCCTGGGAAACAGGTGGTCATTGATAGCAGGTAGAATCCCTGGGAGGACGGCCAATGATATCAAGAACTATTGGAATGCCTGCTTAAGCAAAGGCAAAGCACGTGCCCAAGGAGAGGAAAAGTTACTCGACCAGAGGGTTGTGACACTCAACTCAAATTCTACGAACCCGCACAATCCCATGCATTGTTGCTCCTACTGTGCAACCTTTCCATCTGCTACGAAACCCACTTTCTCCACCACTGTAATCAAGGCTCAACCACGAAACCTATCCAAGAGGCCGCACTGCTTGCCCGAGTTCAACAAAAGCAGTAAAACCAGCCTGAGGGAAACGACGAGCAACACGTATGAGAGGCCTGTCGAGGAGGATAACGATTCATGGTGGAAAAGTCTGTTCGAAGAAGAGACAGTCCAAGAACAAGAACCAGAAAAACAGCCTCTGGAAGGATTTATCAACTCAGTGCATGGGGGCACGGTGACAGGAGAGGCGTCTGCAGGCTTTTGTGGGGAAGGGATAGAAGTACCGAAGGGGGGAGAGGAGAGAGTAGGAAGTGTCAGTTGGGAGGATTTACTCCTGGACCCTAGCCTTATGCGGCTGTTATAA |
| *AcbHLH1* | *>AcbHLH1*  ATGGGCGCCCTGCAGGGTGGCGTCGACGGCGACGGGTGCAGCAGCGGGGCAAATGCTCCCACGCTCCAGCAGATGCTCCAGTCGGCGGTGCAGAGCCTGCAGTGGACCTACAGCCTCTTCTGGCAACTCTGCCCGCAGCAAGGGGCCCTGGTGTGGGCGGAAGGCTACTACAATGGAGCGATAAAGACGAGGAAGGTGGTGCAACCGGTGGAGGCGACGGCGGAGGAGGCGTGCCTCCAGAGGAGCCAGCAGCTGAGGGAGCTCTACGAGTCACTGTCCATGGGGGAGACCAGCCAGCAGCCGGCACGCCGGCCGTGCGCCGCGCTCTCGCCGGAGGACCTCACCGAGTCTGAGTGGTTCTACCTCATGTGCATCTCCTTCTTCTTCCCCCCCGGCGTCGGGTTGCCAGGCAAAGTATTTGAGAGGCAGCAACCTGTGTGGCTTTCAGGGGCAAATGAAGTGGACAGCAAGGTCTTCTCCAGAGCCATTATTGCCAAGGGAGCTCAAATTCAGACTGTCGTTTGCATTCCTCTGGCAGACGGAGTTGTTGAAATCGGCACCACCGATAAGGTGGACGAGGACCCGGCGCTGATGCAGCACGTGCGAAGCTTCTTCACGGGCCACCAGAACTACCCAGCTCAGACGACCACTAAGCCCGCCCTCTCCGAGCACTCCACTTCCAACCCCGTCCCATCCGCCCCCCGCCAGCTCTTCCGCTCACCGTCCCTCCCTGCCATGCCCCCTGCGGCCACGGACTCCTACAGGTACGCCGACGAGGAGGTGGAGGAGGACGACGACGACGATGACGACGACGCCGGGGCAGAGTCCGACTCCGACTCCGAAGCCGACGCCAGAAACGGGGGCAGCGCGGAAGGTGGAGGTGTGTTGCGGCCCTACGCAGTGGCGACAGCTGGGGCGGCCGCCCCGCCGGAAGCCGAGCCCAGCGAGCTGATGCAGATGGAAATGTCGGAGGAGATACGGCTGGGCTCCCCCGACGACTGCTCCAACAACCTCGACGCCGACCTGCAGATGCTGGCCGTGTGCCGGACGGCCAGCGGCCAGTCCGGCGGCCAGGCCGGCGCCGGCCCCGCCTGCCAGGCGTGGCCCCTTCTCCATGACGACGCCACCAACAGCTGCCTTCTTCCATCTTCTGGAGCCACTGCGACGCAAACCATGTCGCAGGAAGACGCCCACTACTCCCAGACGGTGTCCACCATCATGCAGCACAACTCCAGCCGGTGGGTTGACTCCTGCTCCAGTGGGTACCTCGAGCACCCGCGGCAGTCGGCGTTCTCAAGCTGGAGCGGCAGGGGGGAGCACTTCCCAGCGGCTTCCGCGGGAGCCTCGCAGTGGCTGCTCAAGCACGCCCTGCGCAGCGTCCCCTACCTCCACGGCAAGTACAGGGGCGGCGAGAGCTCGCCGAAGCAGTTGAGGGACGCAGAAGGAGGGCAGAGGTTCCGGAAGGGCGGCGCGCTGCAGGACGAGCTGAGCGCCAGCCATGTGTTGGCGGAGAGGAGGAGGAGGGAGAAGCTTAACGAGCGCTTCATCGTCCTCCGCTCTCTGGTTCCATGTGTGACCAAGATGGACAAGGCATCCATATTAGGTGATACAATCGACTACCTAAAACAGCTGCTTAGGCGCATCCAAGAAATGGAGACCCAGATCAAGTTGATGGAGAGTGATAGGAGAGCAAGACCTGTAGAAGCCTCCAAGCCAAACCATAAAGAACTGAGCACCCAAACACATAGCAGTTCCCCACCAGTTGATGCCATCCCACACCTCATCAATGATAAAGTTAGAGTAAGCGGGTCTAATAAGAAGAAACCGAGGGTACTAGGAGGTGGAAGTGGGCCTGCCAAGGCCAAGGCCATGGAGGACACAAGCGTACAAGTTTCCATAATTGAGGCGGATGCCTTGCTGGAGCTACAGTGCCCGAACAGAGATGGCCTCCTGCTCAAGATCATGCAGGCAGTTCACGAGCTTGGGTTGGAGACTACTGCTATCCAATCTTCGTCGGCAGATGGTATCTTTGTGGCAGAAATAAGAGCCAAGGTGAGGGAAAATATTCATGGGAAGAGAGCAAGCATTATGGAAGTAAAGAGGGTGCTTCATCTTCTATTTTCTCAAAGCTTGTGTTAA |
